# Four New Limonoids from the Barks of *Toona ciliata*

**DOI:** 10.1007/s13659-020-00274-w

**Published:** 2020-10-23

**Authors:** Pan-Pan Zhang, Yun-Ge Bu, Shang Xue, Zhi-Rong Cui, Peng-Fei Tang, Jun Luo, Ling-Yi Kong

**Affiliations:** State Key Laboratory of Natural Medicines and Jiangsu Key Laboratory of Bioactive Natural Product Research, School of Traditional Chinese Pharmacy, China Pharmaceutical University, Nanjing, 210009 People’s Republic of China

**Keywords:** Meliaceae, *Toona ciliate*, Limonoids, NO inhibitory effects

## Abstract

**Abstract:**

Four new limonoids, toonayunnanaes F − I (**1** − **4**), and six known compounds (**5** − **10**) were isolated from the barks of *Toona ciliata*. Their structures were elucidated by thoroughly analyzing of NMR and HRMS data, and single-crystal X-ray diffraction of **1**. The oxetane ring moiety in **1** was rare in limonoids and other natural products. Compound **1** showed nitric oxide (NO) inhibitory effect with an IC_50_ 38.45 ± 0.41 µM in lipopolysaccharide (LPS)-activated RAW 264.7 macrophages.

**Graphic Abstract:**

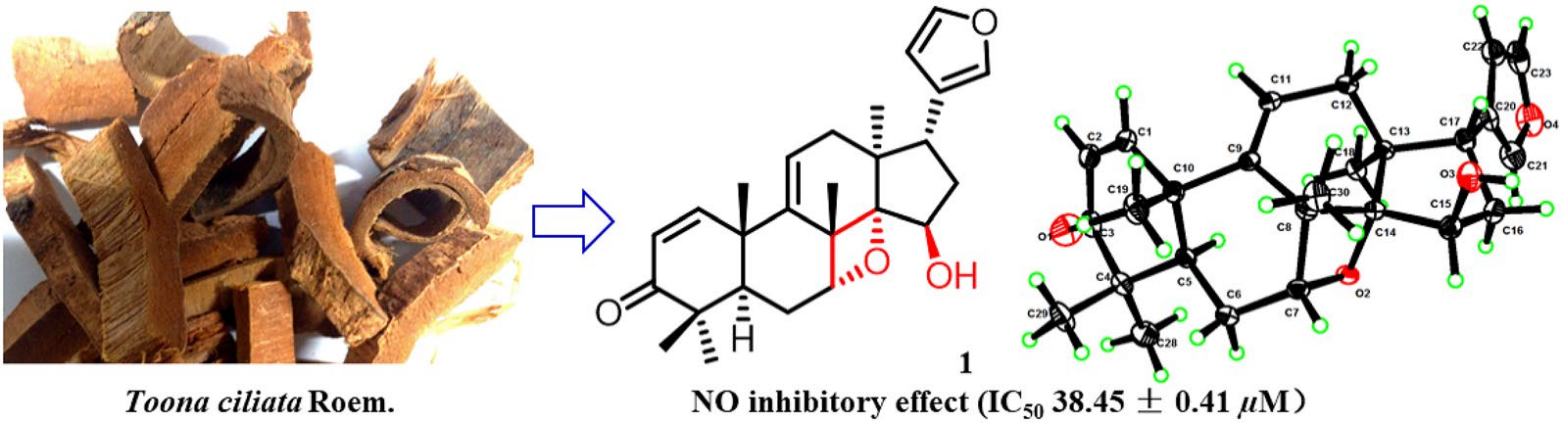

**Electronic supplementary material:**

The online version of this article (10.1007/s13659-020-00274-w) contains supplementary material, which is available to authorized users.

## Introduction

The genus *Toona*, belonging to the family Meliaceae, contains 15 species mainly distributed in the regions of tropical Asia and Africa, of which four species and six varieties grow in China [1]. *Toona ciliata* Roem. var. *ciliata* is a timber tree mainly found in the south of China, such as Yunnan, Sichuan, Guangdong, and Hainan provinces [2]. Its bark has been used in Chinese folk medicine to treat dysentery, fever, and menstrual disorders [3]. Our previous phytochemical investigations on *T. ciliata* var. *henryi* and *T. ciliata* var. *yunnanensis* have led to the isolation of a series of limonoids with potential biological activities such as cytotoxicity, anti-inflammatory, and anti-multidrug resistance (MDR) activities [4–8]. As a part of our continuous research for bioactive limonoids from *Toona* plants, four new limonoids, toonayunnanaes F − I (**1** − **4**), and six known compounds (**5** − **10**) were isolated from the barks of *T. ciliata*. Their structures were elucidated by thoroughly analyzing of NMR and HRMS data, and single-crystal X-ray diffraction of **1**. The oxetane ring moiety in **1** was rare in limonoids and other natural products. Herein, we describe the isolation and structural elucidation of these limonoids as well as their inhibitory effects on NO production in LPS-induced RAW264.7 cells.

## Results and Discussion

**Fig. 1:**
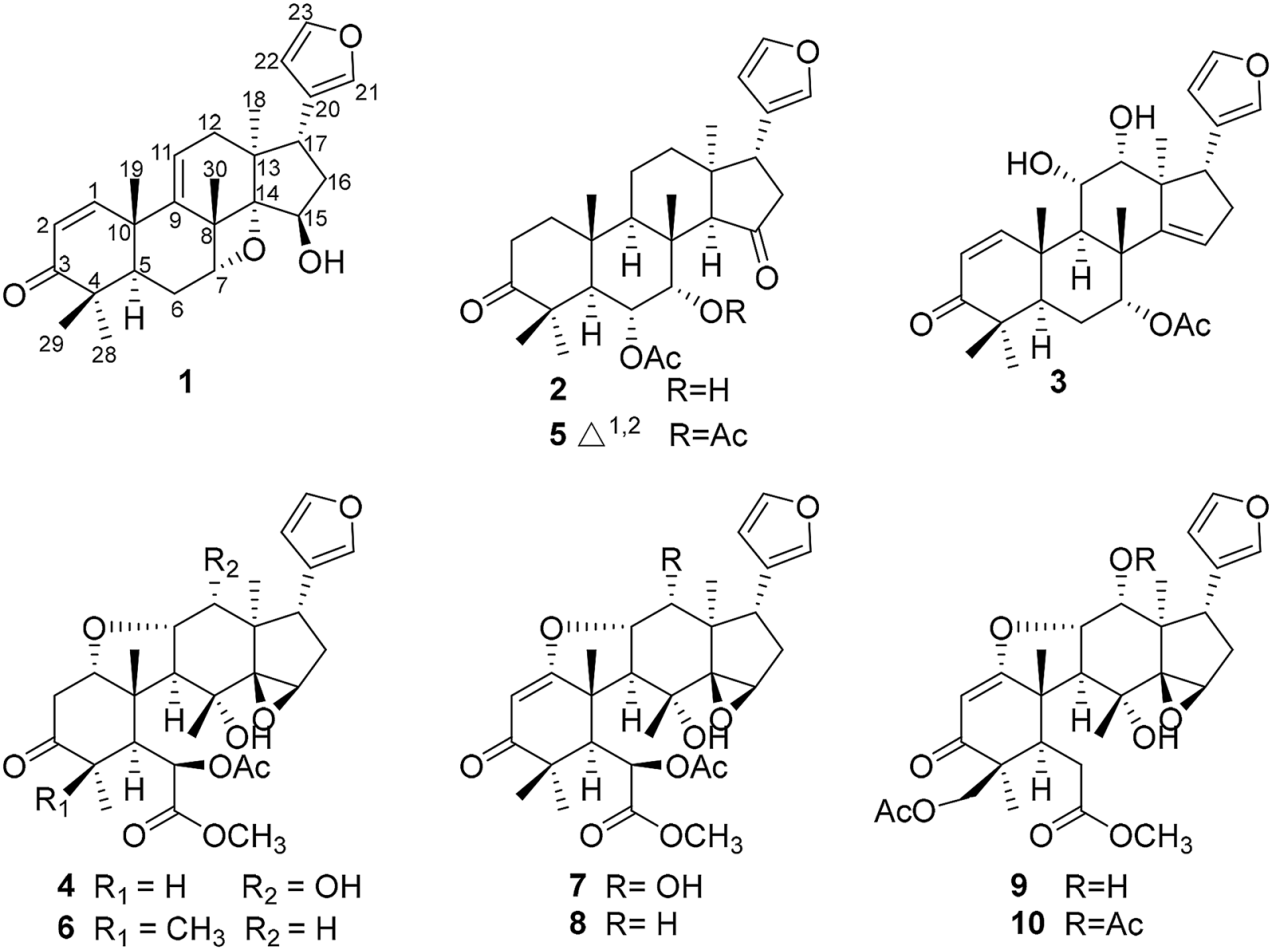
The structures of compounds **1** − **10**

Toonayunnanae F (**1**), colorless crystals, possessed a molecular formula of C_26_H_32_O_4_ according to the HRESIMS ion at *m/z* 409.2376 [M + H]^+^ (calcd. 409.2373). The ^1^H and ^13^C NMR data of **1** (Table 1) showed characteristic resonances for a *β*-substituted furan ring (*δ*_H_ 7.35, 7.16, 6.20; *δ*_C_ 111.2, 124.7, 139.4, 142.8), an *α,β*-unsaturated ketone (*δ*_H_ 7.08 d, *J* = 10.4 Hz, 5.98 d, *J* = 10.4 Hz; *δ*_C_ 125.7, 155.0, 204.3), and five singlet methyl groups (*δ*_H_ 0.54, 1.05, 1.11, 1.29, 1.54). Those observations together with 26 carbon resonances and 11 indices of hydrogen deficiency, suggested that **1** might be a ring-intact limonoid with an *α,β-*unsaturated carbonyl moiety in A ring [8–10] (Fig. 1).

**Table 1:** ^1^H and ^13^C NMR data of compounds **1** − **4** in CDCl_3_ (*δ* in ppm, *J* in Hz)

No.	1^a^		2^b^		3^a^		4^a^	
	*δ*_H_	*δ*_C_	*δ*_H_	*δ*_C_	*δ*_C_	*δ*_C_	*δ*_H_	*δ*_C_
1	7.08 d (10.4)	155.0	1.90 m 1.77 m	39.1	8.00 d (10.4)	160.6	3.98 t (5.7)	88.0
2	5.98 d (10.4)	125.7	2.76 m 2.31 m	32.9	5.84 d (10.4)	124.0	2.71 d (5.7)	42.7
3		204.3		217.8		204.3		210.1
4		44.2		46.4		44.3	2.59 m	41.8
5	2.32 dd (13.2, 4.0)	44.7	2.64 d (12.1)	46.7	2.28 dd (13.3, 2.6)	46.0	2.61 s	51.2
6	1.90 m 1.80 m	26.3	5.31 dd (12.1, 2.3)	72.2	1.98 m 1.83 m	23.9	5.93 s	71.4
7	4.82 d (3.5)	81.5	3.90 br s	72.1	5.27 br s	73.9		170.8
8		47.1		42.0		42.2		76.2
9		148.5	1.41 d (10.4)	48.6	2.37 d (8.3)	48.8	2.90 d (12.0)	54.0
10		41.4		38.3		40.8		48.0
11	5.66 dd (8.0, 4.2)	121.4	1.56 td (13.5, 3.1) 1.51 m	17.9	4.30 dd (8.3, 6.1)	67.8	4.06 dd (12.0, 4.7)	79.3
12	2.08 m	36.1	1.94 m 1.21 dd (13.5, 4.9)	34.5	4.03 d (6.1)	77.1	4.23 d (4.7)	71.5
13		51.6		42.1		51.4		47.2
14		96.8	2.84 s	60.8		157.5		74.2
15	4.62 br s	77.7		221.2	5.49 br s	121.1	3.50 s	54.8
16	1.98 m 1.87 m	37.9	2.50 d (10.2)	43.4	2.49 dd (15.2, 10.9) 2.41 m	36.3	2.32 dd (13.6, 6.6) 1.84 dd (13.6, 11.0)	31.3
17	3.27 dd (13.3, 5.1)	43.1	3.46 t (10.2)	37.8	3.09 dd (10.9, 7.6)	50.3	2.80 dd (11.0, 6.6)	41.3
18	0.54 s	19.1	0.78 s	27.7	0.99 s	14.8	1.02 s	14.9
19	1.29 s	20.9	0.84 s	18.2	1.31 s	20.5	1.27 s	18.1
20		124.7		122.9		125.3		123.0
21	7.16 s	139.4	7.26 s	140.3	7.39 s	140.4	7.18 s	139.5
22	6.20 s	111.2	6.27 s	110.9	6.46 s	111.7	6.38 s	110.0
23	7.35 s	142.8	7.39 s	143.0	7.40 s	143.2	7.38 s	143.2
28	1.11 s	24.8	1.24 s	31.3	1.09 s	26.9	1.06 d (5.8)	13.0
29	1.05 s	21.6	1.05 s	19.6	1.08 s	21.5		
30	1.54 s	22.3	1.14 s	17.2	1.24 s	30.6	1.24 s	23.0
6-Oac			2.13 s	21.8			2.20 s	21.1
6-Oac				169.8				170.3
7-OCH_3_							3.72 s	52.9
7-OAc					1.98 s	21.3		
7-OAc						170.2		

^a^500 MHz for ^1^H NMR, 125 MHz for ^13^C NMR

^b^600 MHz for ^1^H NMR, 150 MHz for ^13^C NMR

**Fig. 2:**
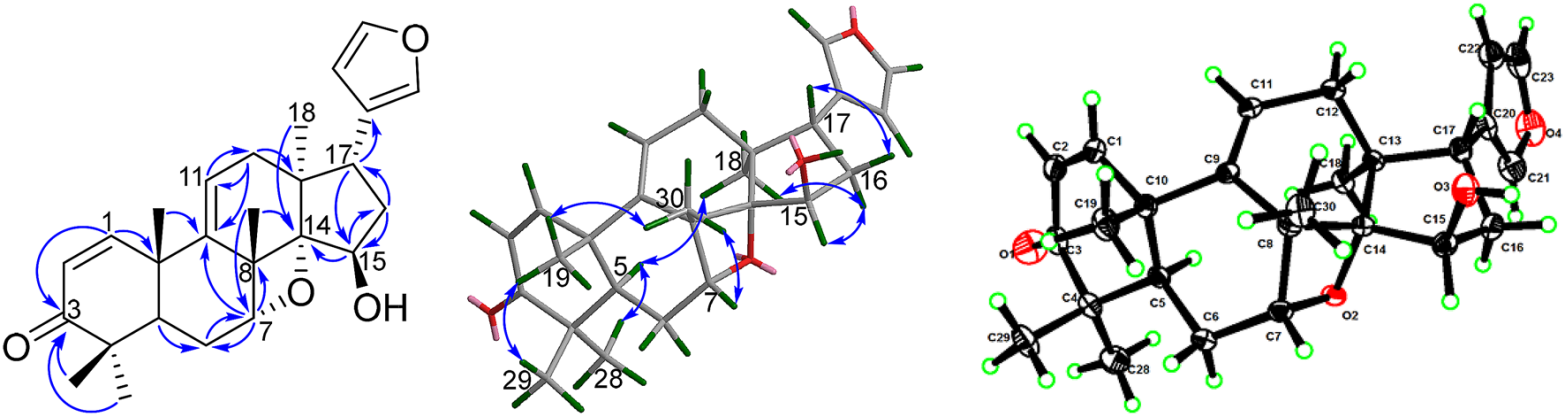
The key HMBC and ROESY correlations and X-ray crystallographic structure of compound **1**

The double bond between C-9 and C-11 was confirmed by the correlations of H_3_-19 (*δ*_H_ 1.29) and H_3_-30 to C-9 (*δ*_C_ 148.5); of H-11 (*δ*_H_ 5.66) to C-12 (*δ*_C_ 36.1); and of H_2_-12 (*δ*_H_ 2.08) to C-9 and C-11 (*δ*_C_ 121.4) in the HMBC spectrum. The HMBC correlations (Fig. 2) from H-7 (*δ*_H_ 4.82) to C-8 (*δ*_C_ 47.1) and C-6 (*δ*_C_ 26.3); from H_3_-30 (*δ*_H_ 1.54) to C-7 (*δ*_C_ 81.5) and C-14 (*δ*_C_ 96.8); from H_3_-18 (*δ*_H_ 0.54) and H-15 (*δ*_H_ 4.62) to C-14; and from H_2_-16 and H-17 to C-15 (*δ*_C_ 77.7) indicated that C-7, C-14, and C-15 were all oxygenated. The significantly deshielded chemical shift of C-14 and the indices of hydrogen deficiency suggested the presence of an oxetane ring between C-7 and C-14 [8, 11, 12] and a hydroxy group at C-15, similar to that in ciliatasecone S [8]. Therefore, the 2D structure of **1** with a rare oxetane ring was constructed. The ROESY cross-peaks of H_3_-19/H_3_-29, H_3_-19/H_3_-30, and H_3_-30/H-7 (Fig. 2) revealed the *β*-orientations of Me-19, Me-29, Me-30, and H-7. The *α*-orientations of H-5, Me-18, and Me-28 were determined by the ROESY cross-peaks of H-5/H_3_-28 and H-5/H_3_-18. The cross-peaks of H_3_-18/H-16 (*δ*_H_ 1.98), H-16 (*δ*_H_ 1.98)/H-15, and H-16 (*δ*_H_ 1.87)/H-17 indicated the *β*-orientation of 15-OH and the *α*-orientation of furan ring. A single-crystal X-ray crystallographic diffraction experiment with Cu Kα radiation of **1** (Fig. 2) unambiguously determined the *α*-orientation of the oxetane ring moiety and the absolute configuration as 5*R*, 7*R*, 8*S*, 10*R*, 13*S*, 14*S*, 15*R*, and 17*S*. The structure of compound **1** was thus established as shown.

**Fig. 3:**
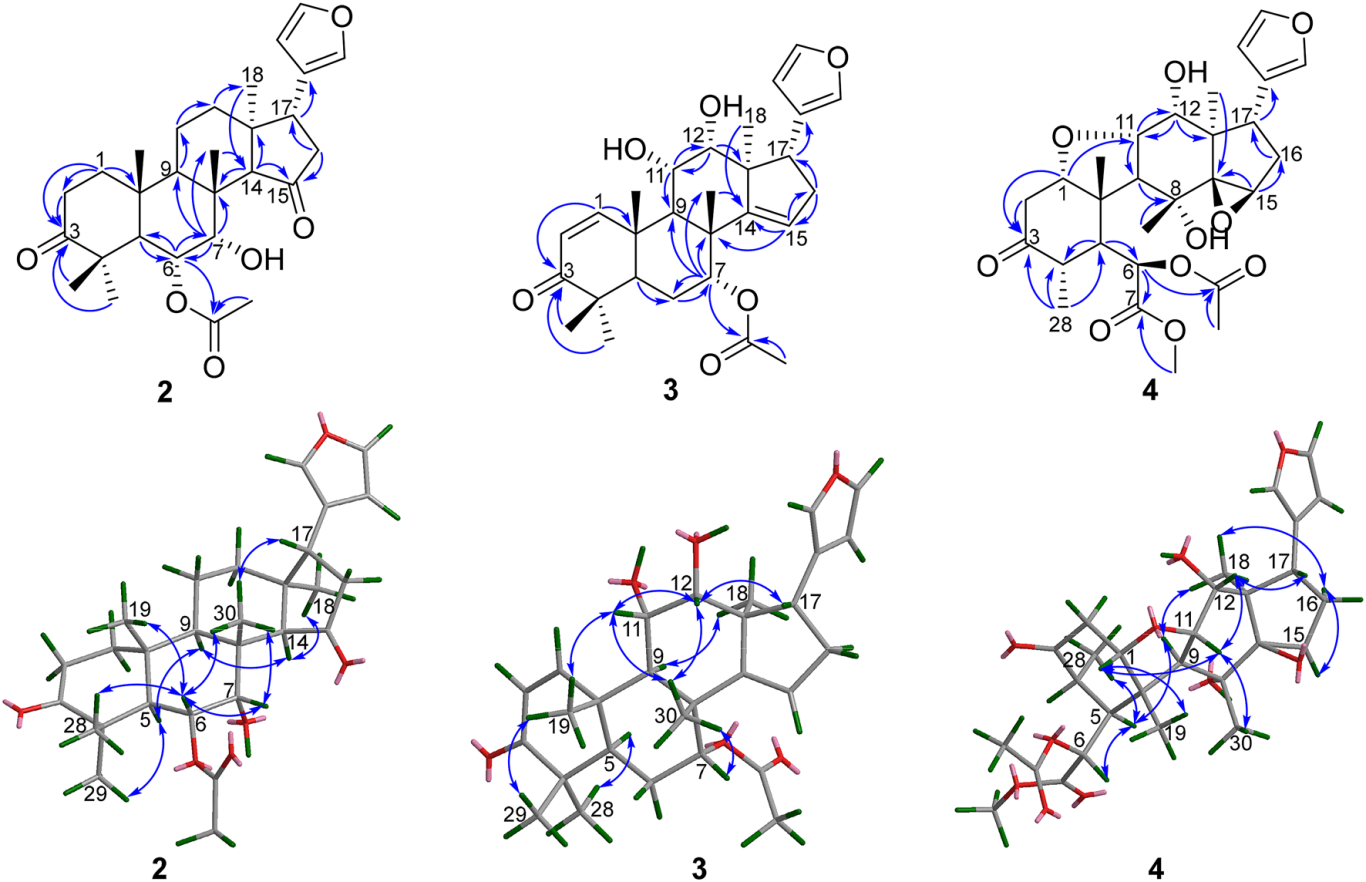
The key HMBC and ROESY correlations of compounds **2** − **4**

The molecular formula of toonayunnanae G (**2**) was established as C_28_H_38_O_6_ based on the HRESIMS ion at *m/z* 493.2551 [M + Na]^+^ (calcd. 493.2561) and its ^13^C NMR data. Apart from the signals for an acetoxy substituent, the remaining 26 carbon resonances observed in the 1D NMR spectra indicated that **2** might also be a ring-intact limonoid [8–10]. Comparison of its NMR data (Table 1) with those of the known toonaciliatone F (**5**) [13] revealed that the main differences were the absence of a double bond and an acetoxy group. The chemical shift of C-3 (*δ*_C_ 217.8) implied the presence of a keto carbonyl rather than an *α,β*-unsaturated ketone moiety in ring A, which was confirmed by the HMBC correlations of H_2_-1 (*δ*_H_ 1.90, 1.77), H_2_-2 (*δ*_H_ 2.76, 2.31), H_3_-28, and H_3_-29 to C-3. The HMBC correlations of H-6 (*δ*_H_ 5.31) to C-5, C-7 (*δ*_C_ 72.2), and an ester carbonyl carbon (*δ*_C_ 169.8) and of H-7 (*δ*_H_ 3.90) to C-6 (*δ*_C_ 72.1), C-8, C-9, and C-30 (Fig. 3) allowed the assignment of an acetoxyl at C-6 and a hydroxy group at C-7. The relative configuration of **2** was deduced to be identical to that of **5** by a ROESY experiment. The ROESY cross-peaks of H_3_-19/H-6, H_3_-29/H-6, H_3_-30/H-6, H-6/H-7, and H-7/H_3_-30 revealed the *α*-orientations of 6-OAc and 7-OH. Therefore, the structure of **2** was assigned as depicted.

Toonayunnanae H (**3**) gave a molecular formula of C_28_H_36_O_6_ as determined from HRESIMS ion at *m/z* 469.2577 [M + H]^+^ (calcd. 469.2585). The ^1^H and ^13^C NMR spectroscopic data showed many similarities to those of the known 6*α*-acetoxyazadirone [13], except for the absence of an acetoxy group and the presence of a hydroxy group. The HMBC correlations of H-7 (*δ*_H_ 5.27) to C-6, C-8, C-9, C-30, and an ester carbonyl carbon (*δ*_C_ 170.2) indicated that an acetoxy group was located at C-7. The hydroxy groups at C-11 and C-12 were revealed by the HMBC correlations (Fig. 3) of H-11 (*δ*_H_ 4.30) to C-9 and C-12 (*δ*_C_ 77.1) and of H-12 (*δ*_H_ 4.03) to C-11 (*δ*_C_ 67.8) and C-13. The ROESY cross-peaks of H_3_-30/H-7, H_3_-30/H-11, H-11/H-12, and H-12/H-17 (Fig. 3) suggested the *α*-orientations of 7-OAc, 11-OH, and 12-OH. Therefore, the structure of **3** was determined as shown.

The clearly diagnostic signals (Table 1) for a *β*-substituted furan moiety, five characteristic singlet methyl groups in the 1D NMR spectra, and the correlations of a methoxy group (*δ*_H_ 3.72) with an ester carbonyl carbon (*δ*_C_ 170.8) in the HMBC spectrum suggested that toonayunnanae I (**4**) was likely a B-*seco* limonoid with a C6-C7-methyl ester appendage [5, 8, 14–16], similar to toonayunnanin G (**6**) [15]. Comparison of the 1D NMR data (Table 1) of **4** with those of **6** revealed that the major difference was the presence of a doublet methyl signal (*δ*_H_ 1.06, 3H, d, *J* = 5.8 Hz), which was ascribed to Me-28 at C-4 based on its HMBC correlations (Fig. 3) with C-3 (*δ*_C_ 210.1), C-4 (*δ*_C_ 41.8), and C-5 (*δ*_C_ 51.2). Therefore, **4** possessed a B-*seco*-29-nor-limonoid skeleton similar to that of ciliatasecone N [8]. The HMBC correlations of H-12 (*δ*_H_ 4.23) to C-11 (*δ*_C_ 79.3) and C-13 indicated that a hydroxy group was located at C-12. The relative configuration of **4** was deduced to be identical to that of **6** by a ROESY experiment. The *α*-orientations of 12-OH and Me-28 in **4** was inferred from the ROESY cross-peaks (Fig. 3) of H-11/H-12, H-12/H-17, and H_3_-28/H-5. Accordingly, the structure of **4** was elucidated as shown.

By comparing their ^1^H and ^13^C NMR spectroscopic data with those reported in literatures, six known compounds were identified as toonaciliatone F (**5**) [13], toonayunnanin G (**6**) [15], toonacilianin G (**7**) [16], toonaciliatin P (**8**) [17], toonaciliatin C (**9**) [18], and toonacilianin H (**10**) [16], respectively.

Additionally, the inhibitory effects on NO generation in LPS-activated RAW 264.7 macrophages of compounds **1** − **4** were evaluated at the concentrations of 50 μM and below. Compound **1** showed NO inhibitory effect with an IC_50_ 38.45 ± 0.41 µM.

## Experimental

### General Experimental Procedures

Optical rotations were measured on a JASCO P-1020 automatic digital polarimeter at room temperature. IR spectra were recorded on a Bruker Tensor 27 spectrometer using KBr pellets. UV spectra were recorded on a Shimadzu UV-2450 spectrophotometer (Shimadzu, Tokyo, Japan). High-resolution electrospray ionization mass spectrometry (HRESIMS) was obtained on an Agilent 6529B Q-TOF mass instrument using electrospray ionization. The 1D and 2D nuclear magnetic resonance (NMR) spectra were obtained on Bruker AVANCE III 500 MHz or Bruker AVIIIHD 600 MHz spectrometers in CDCl_3_ with TMS as an internal standard. Analytical HPLC was conducted on an Agilent 1260 infinity system equipped with a DAD-UV detector. Preparative HPLC was carried out using a Shimadzu LC-6A system (Shimadzu, Tokyo, Japan) equipped with a Shim-pack RP-C_18_ column (20 × 200 mm, i.d. 10 μm, Shimadzu, Tokyo, Japan) with a flow rate of 10.0 mL/min, detected by a binary channel UV detector. Silica gel (200–300 mesh, Qingdao Haiyang Chemical Co. Ltd., China), MCI (Mitsubishi, Japan), and RP-C_18_ silica (40–63 μm, FuJi, Japan) were used for column chromatography.

### Plant Material

The air-dried bark of *Toona ciliata* Roem. var. *ciliata* was collected in Baoshan, Yunnan Province, China, in August 2018. The plant material was identified by professor Mian Zhang of the research Department of Pharmacognosy, China Pharmaceutical University. A voucher specimen (no. 2018-TC) was deposited in the Department of Natural Medicinal Chemistry, China Pharmaceutical University.

### Extraction and Isolation

The air-dried and powder bark of *T. ciliata* (29 kg) was extracted with 95% EtOH three times (3 × 6.5 L) under reflux. The concentrated extract (3.1 kg) was suspended in H_2_O, and then partitioned with petroleum ether (PE) and dichloromethane (DCM), successively. The DCM extract (100.0 g) was subjected to a silica gel column (200 − 300 mesh) eluted with a PE-EtOAc mixture (100:0 − 100:1 − 100:2 − 100:3 − 100:5 − 10:1 − 5:1, v/v) in a step gradient to obtain eight major fractions (A − H). Fraction C (20.0 g) was loaded onto an ODS column eluted with a mixture of MeOH-H_2_O from 40 to 70% to afford four fractions (C1 − C4). Fraction C4 (3.3 g) was further applied to an ODS column (30% − 60% ACN-H_2_O) to give four subfractions (C4a − C4d). Fraction C4a (374 mg) was separated by preparative HPLC with 60% MeOH-H_2_O to yield **1** (4 mg) and **2** (11 mg). Similarly, fraction C4c (582 mg) afforded **3** (5.3 mg), **5** (14 mg), and **8** (10.3 mg) by preparative HPLC (50% ACN-H_2_O). Fraction D (15 g) was subjected to an MCI column (50% − 70% MeOH-H_2_O) to give three subfractions (D1 − D3). Fraction D2 (5.5 g) was separated by an ODS MPLC (40% − 60% ACN-H_2_O) to afford five subfractions (D2a − D2e), and fraction D2b (539 mg) was purified by preparative HPLC with MeOH-H_2_O (60% MeOH-H_2_O) to give **6** (5.2 mg) and **9** (14 mg). Using the same purification procedures, fraction D3 (3.3 g) was further fractionated by ODS column chromatography (40% − 60% MeOH-H_2_O), and the subfraction D3c (483 mg) was purified by preparative HPLC with 45% ACN‑H_2_O to give **4** (2.2 mg), **7** (34.1 mg), and **10** (45 mg).

Toonayunnanae F (**1**): colorless crystals (MeOH-H_2_O); [*α*]_D_^24^ + 7.6 (*c* 0.1, MeOH); UV (MeOH) *λ*_max_ (log *ε*) 218 (5.30) nm; IR (KBr) *ν*_max_ 3553, 2986, 2931, 2859, 1669, 1455, 1387, 1158, 1025 cm^−1^; ^1^H and ^13^C NMR data, see Table 1; HRESIMS *m/z* 409.2376 [M + H]^+^ (calcd. for C_26_H_33_O_4_, 409.2373).

Toonayunnanae G (**2**): white amorphous powder; [*α*]_D_^24^ + 9.2 (*c* 0.1, MeOH); UV (MeOH) *λ*_max_ (log *ε*) 208 (3.95) nm; IR (KBr) ν_max_ 3409, 2964, 1724, 1384, 1247, 1028 cm^−1^; ^1^H and ^13^C NMR data, see Table 1; HRESIMS *m/z* 493.2551 [M + Na]^+^ (calcd. for C_28_H_38_NaO_6_, 493.2561).

Toonayunnanae H (**3**): white amorphous powder; [*α*]_D_^24^ + 7.6 (*c* 0.1, MeOH); UV (MeOH) *λ*_max_ (log *ε*) 208 (3.55) nm; IR (KBr) *ν*_max_ 3421, 2977, 1732, 1664, 1249, 1027 cm^−1^; ^1^H and ^13^C NMR data, see Table 1; HRESIMS *m/z* 469.2577 [M + H]^+^ (calcd. for C_28_H_37_O_6_, 469.2585).

Toonayunnanae I (**4**): white amorphous powder; [*α*]_D_^24^ − 30.2 (*c* 0.1, MeOH); UV (MeOH) *λ*_max_ (log *ε*) 208 (4.02) nm; IR (KBr) *ν*_max_ 3466, 2954, 1747, 1717, 1226, 1026 cm^−1^; ^1^H and ^13^C NMR data, see Table 1; HRESIMS *m/z* 550.2631 [M + NH_4_]^+^ (calcd. for C_28_H_40_NO_10_, 550.2647).

### Anti-inflammatory Activities

The new compounds (**1** − **4**) were evaluated for their inhibitory effects on NO production in LPS-activated RAW 264.7 macrophages as described in the literature [19]. Briefly, RAW 264.7 cells (6 × 10^6^ cells/mL) were seeded in 96-well plates and treated with different concentrations of tested compounds for 1 h, and 1.0 μg/mL LPS solution was subsequently added to stimulate the cells for 18 h. NO level was evaluated by measuring the standard of accumulated nitrite in cell supernatants with the reagent of Griess. N-Monomethyl-L-arginine Monoacetate (L-NMMA) was used as a positive control (IC_50_ = 42.38 ± 0.72 μM).

## Electronic supplementary material

Below is the link to the electronic supplementary material.

Supplementary file1 (PDF 1690 kb)
