# Supplementary material for: Four New Limonoids from the Barks of Toona ciliata
Source: Nat Prod Bioprospect. 2020 Oct 23;11(1):81–6. doi: 10.1007/s13659-020-00274-w (PMC7933302; doi:10.1007/s13659-020-00274-w)
Supplement: Supplementary file 1 — Supplementary file1 (PDF 1690 kb) [file 13659_2020_274_MOESM1_ESM.pdf]

Four new limonoids from the barks of *Toona ciliata*

Pan-Pan Zhang · Yun-Ge Bu · Shang Xue · Zhi-Rong Cui · Peng-Fei Tang · Jun Luo* · Ling-Yi Kong*

State Key Laboratory of Natural Medicines and Jiangsu Key Laboratory of Bioactive Natural Product Research, School of Traditional Chinese Pharmacy, China Pharmaceutical University, Nanjing 210009, People's Republic of China

*Corresponding authors:

E-mail addresses: cpu_lykong@126.com (Ling-Yi Kong); luojun@cpu.edu.cn (Jun Luo)

Table of content

Figure S1. The ^{1}H NMR (500 MHz, $CDCl_3$) spectrum of compound **1**.

Figure S2. The ^{13}C NMR (125 MHz, $CDCl_3$) spectrum of compound **1**.

Figure S3. The HSQC ($CDCl_3$) spectrum of compound **1**.

Figure S4. The HMBC ($CDCl_3$) spectrum of compound **1**.

Figure S5. The ROESY ($CDCl_3$) spectrum of compound **1**.

Figure S6. The UV spectrum of compound **1**.

Figure S7. The IR spectrum of compound **1**.

Figure S8. The HR-ESI-MS spectrum of compound **1**.

Figure S9. The ^{1}H NMR (600 MHz, $CDCl_3$) spectrum of compound **2**.

Figure S10. The ^{13}C NMR (150 MHz, $CDCl_3$) spectrum of compound **2**.

Figure S11. The HSQC ($CDCl_3$) spectrum of compound **2**.

Figure S12. The HMBC ($CDCl_3$) spectrum of compound **2**.

Figure S13. The ROESY ($CDCl_3$) spectrum of compound **2**.

Figure S14. The UV spectrum of compound **2**.

Figure S15. The IR spectrum of compound **2**.

Figure S16. The HR-ESI-MS spectrum of compound **2**.

Figure S17. The ^{1}H NMR (500 MHz, $CDCl_3$) spectrum of compound **3**.

Figure S18. The ^{13}C NMR (125 MHz, $CDCl_3$) spectrum of compound **3**.

Figure S19. The HSQC ($CDCl_3$) spectrum of compound **3**.

Figure S20. The HMBC ($CDCl_3$) spectrum of compound **3**.

Figure S21. The ROESY ($CDCl_3$) spectrum of compound **3**.

Figure S22. The UV spectrum of compound **3**.

Figure S23. The IR spectrum of compound **3**.

Figure S24. The HR-ESI-MS spectrum of compound **3**.

Figure S25. The ^{1}H NMR (500 MHz, $CDCl_3$) spectrum of compound **4**.

Figure S26. The ^{13}C NMR (125 MHz, $CDCl_3$) spectrum of compound **4**.

Figure S27. The HSQC ($CDCl_3$) spectrum of compound **4**.

Figure S28. The HMBC ($CDCl_3$) spectrum of compound **4**.

Figure S29. The ROESY ($CDCl_3$) spectrum of compound **4**.

Figure S30. The UV spectrum of compound **4**.

Figure S31. The IR spectrum of compound **4**.

Figure S32. The HR-ESI-MS spectrum of compound **4**.

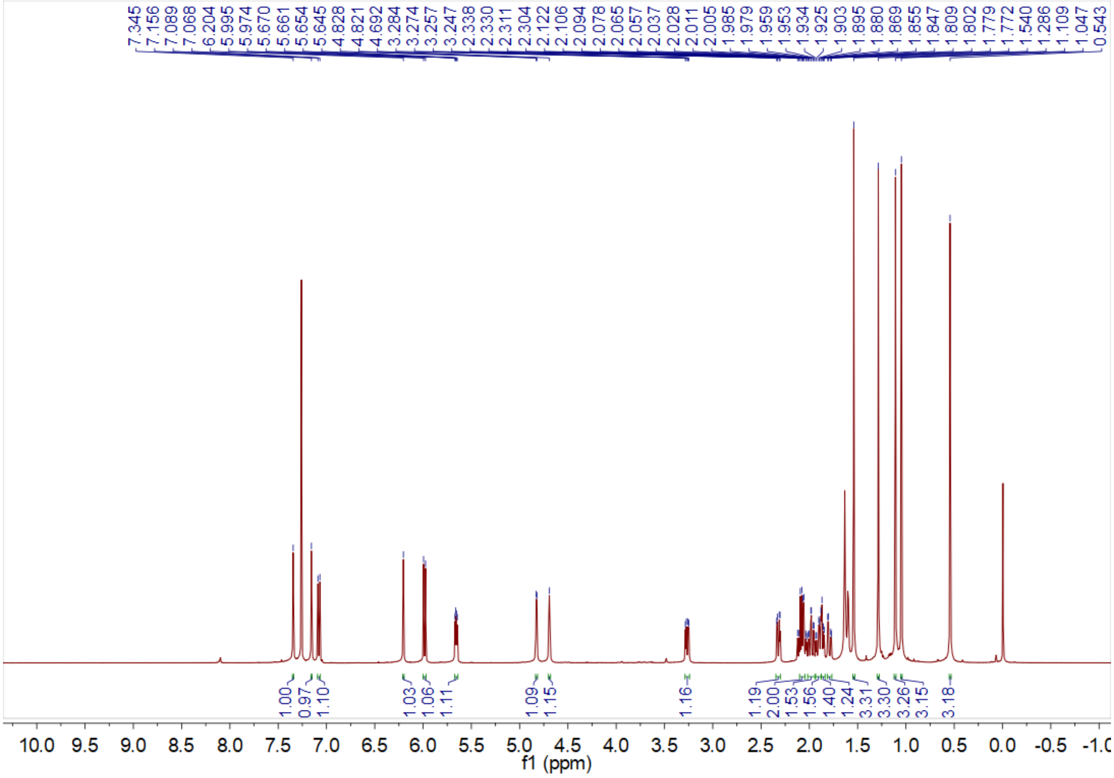

Figure S1. The ^{1}H NMR (500 MHz, $CDCl_3$) spectrum of compound **1**.

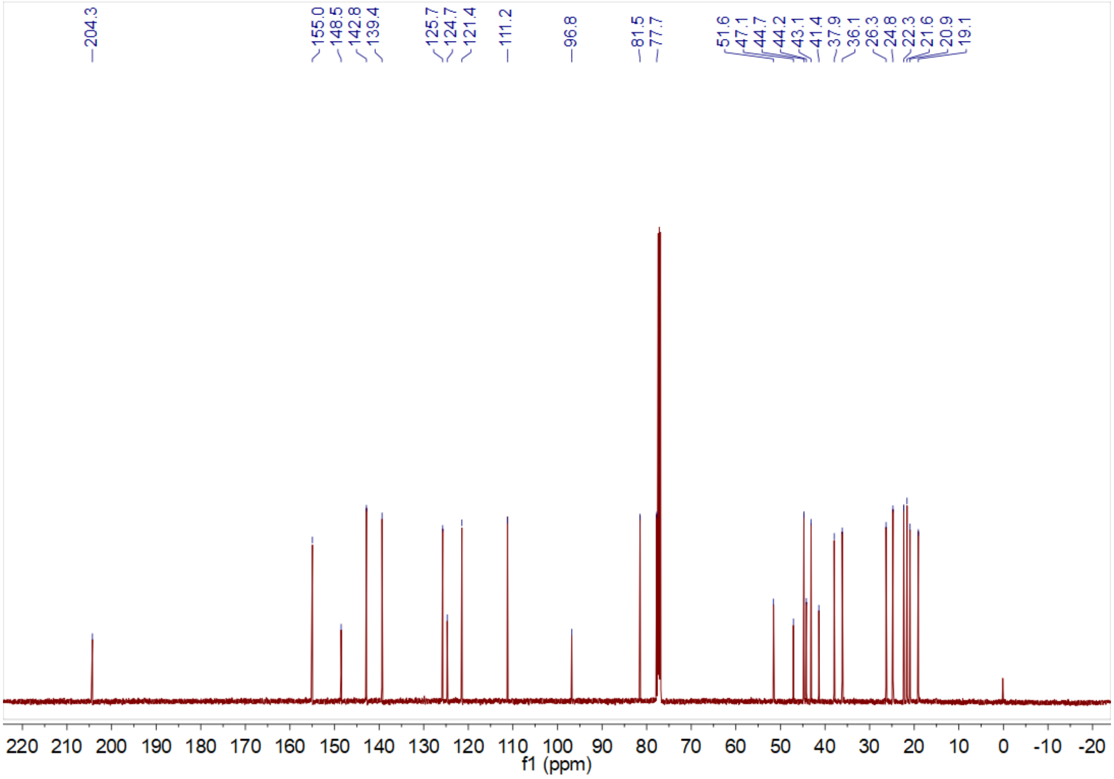

Figure S2. The ^{13}C NMR (125 MHz, $CDCl_3$) spectrum of compound **1**.

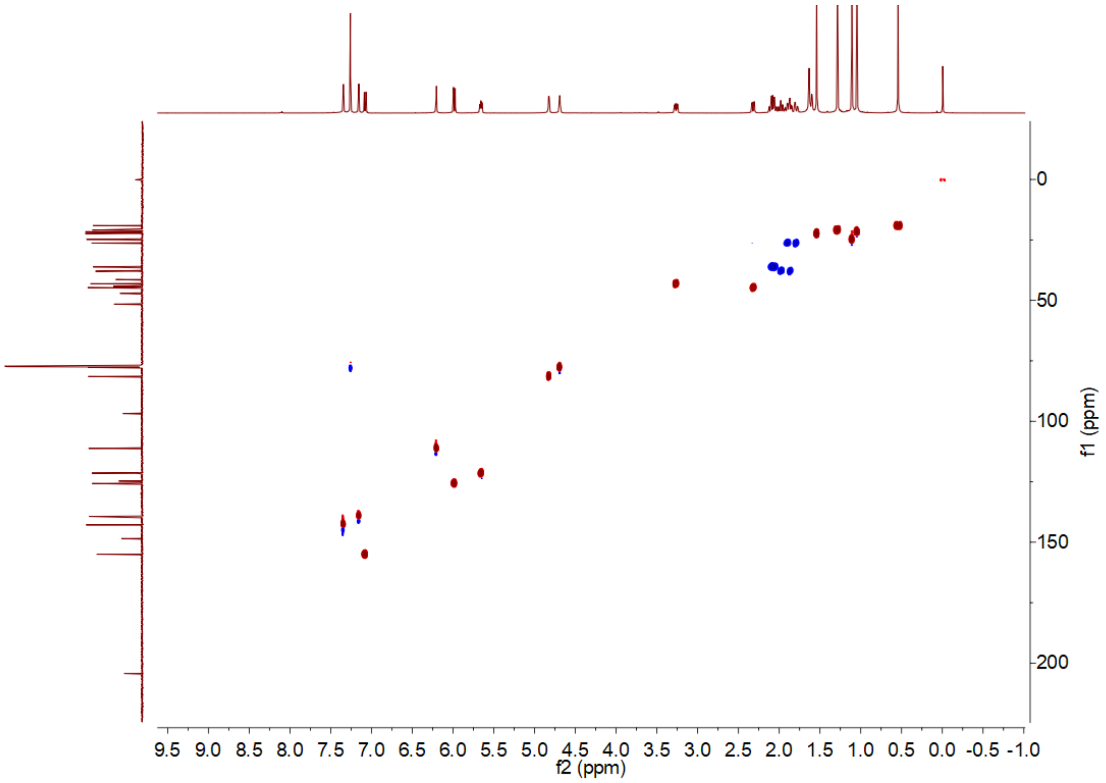

Figure S3. The HSQC ($CDCl_3$) spectrum of compound **1**.

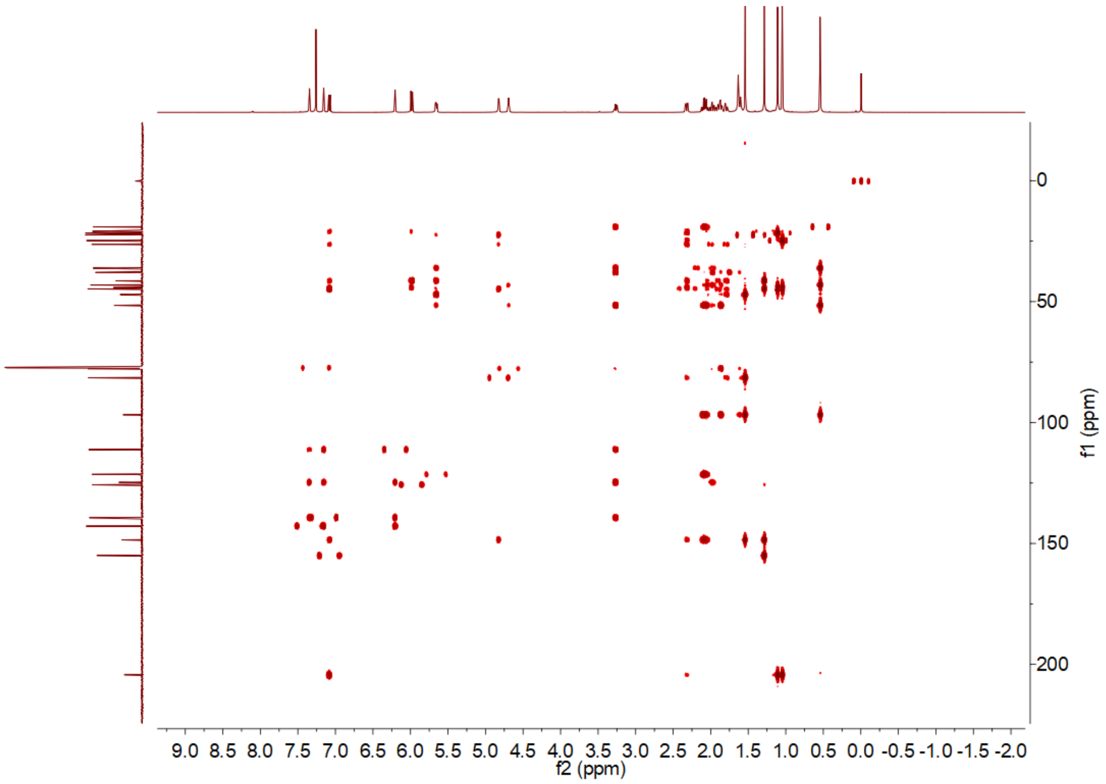

Figure S4. The HMBC ($CDCl_3$) spectrum of compound **1**.

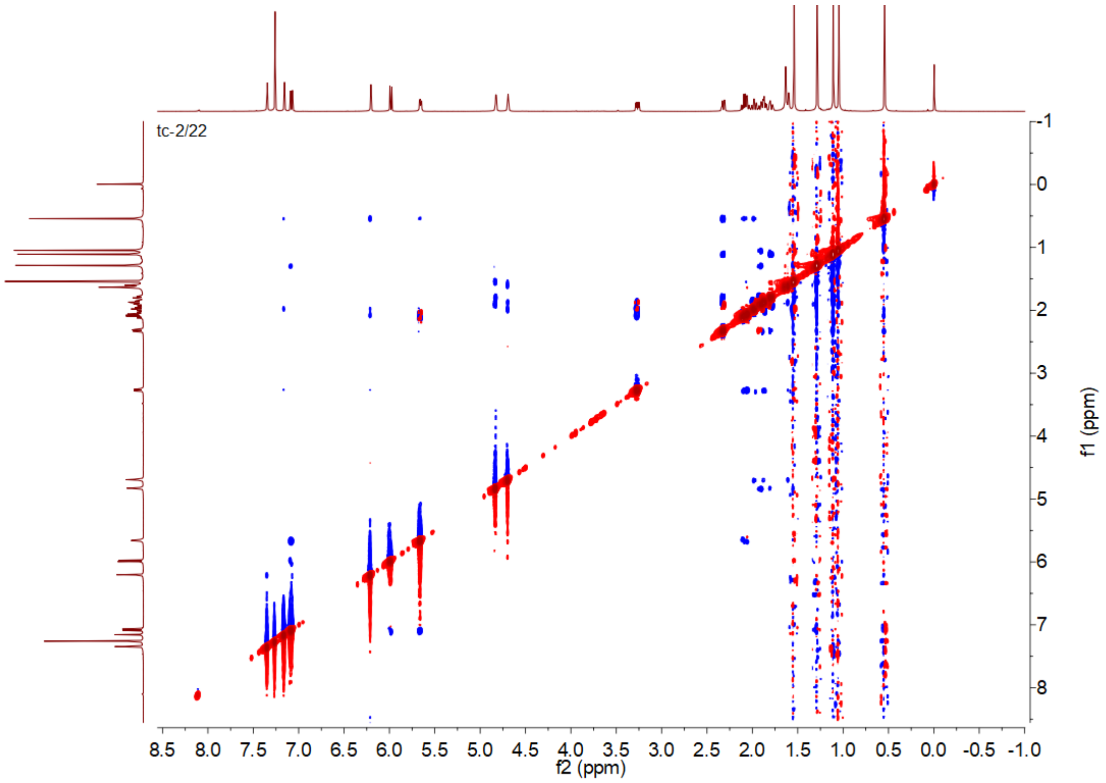

Figure S5. The ROESY ($CDCl_3$) spectrum of compound **1**.

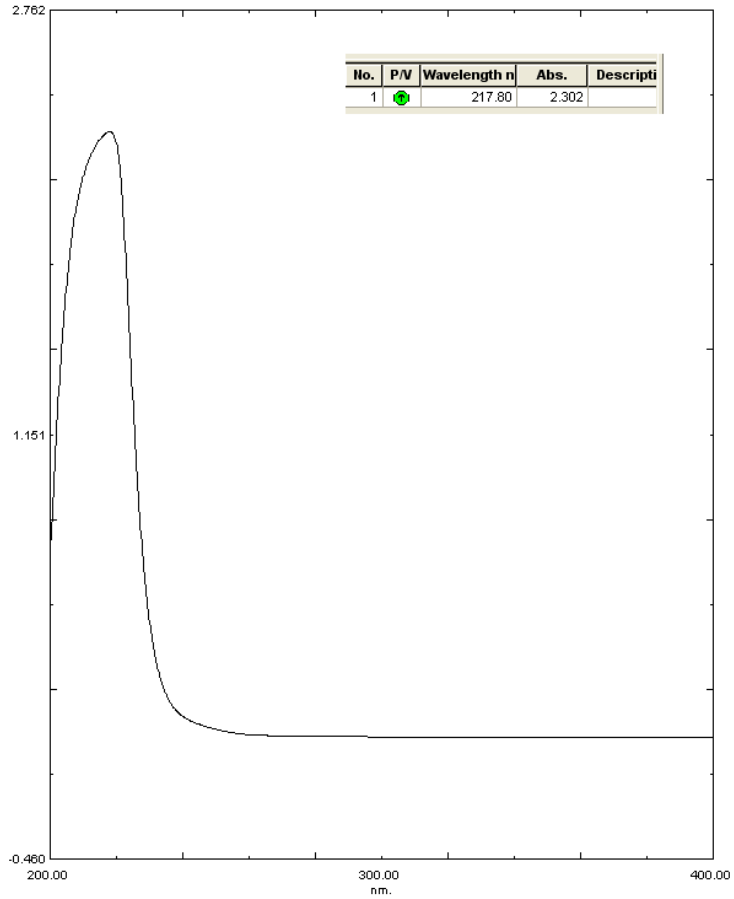

Figure S6. The UV spectrum of compound **1**.

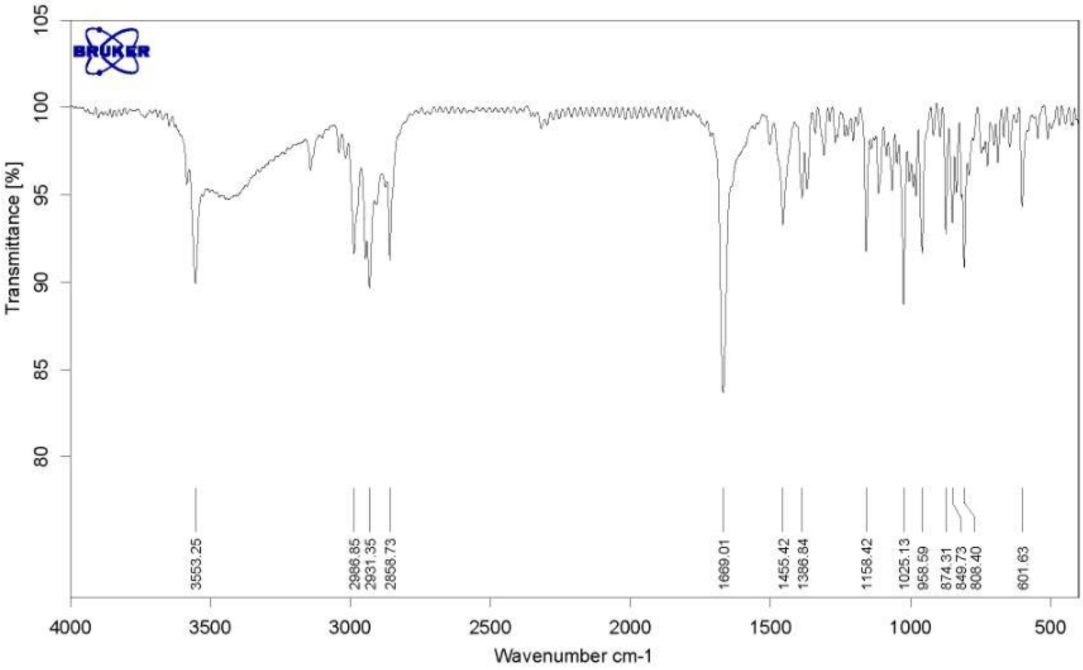

Figure S7. The IR spectrum of compound **1**.

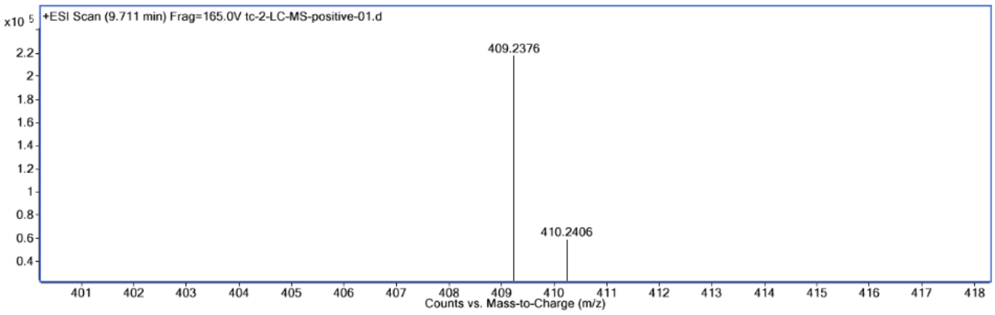

Elemental Composition Calculator

**Target m/z:**	409.2376	**Result type:**	Positive ions	**Species:**	$[M+H]^+$
**Elements:**		C (0-80); H (0-120); O (0-30)			
**Ion Formula**		**Calcalated m/z**		**PPM Error**	
C26H33O4		409.2373		-0.74	

Figure S8. The HR-ESI-MS spectrum of compound **1**.

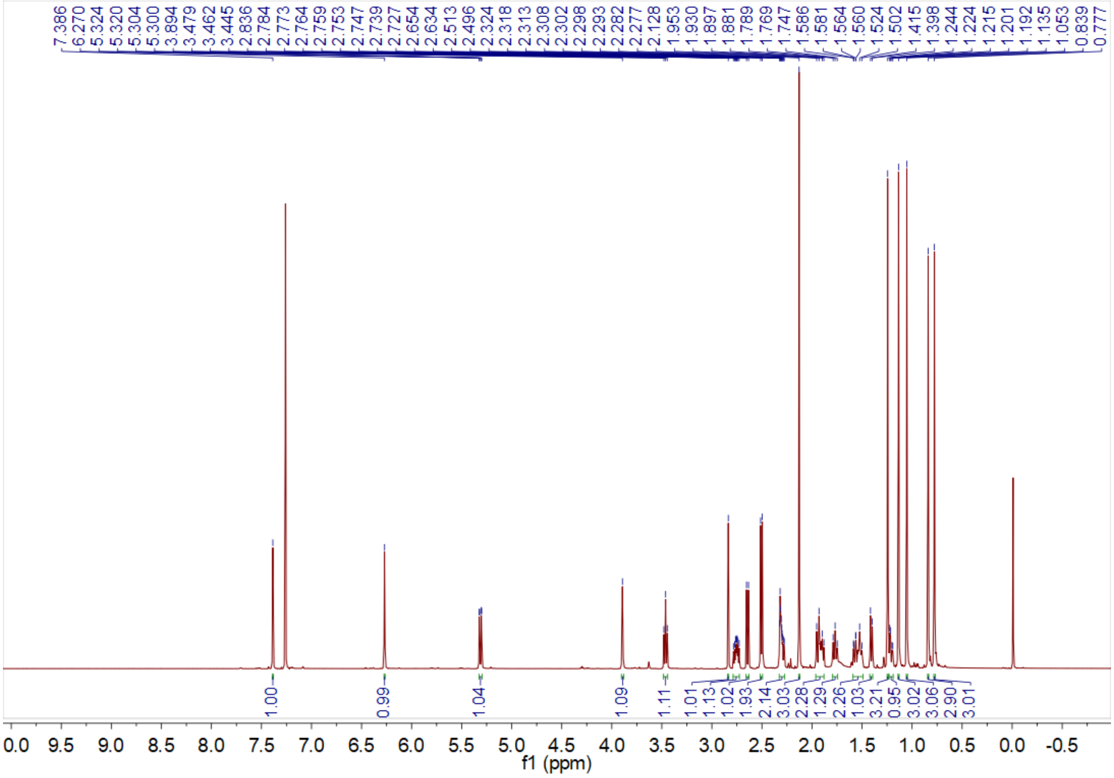

Figure S9. The ^{1}H NMR (600 MHz, $CDCl_3$) spectrum of compound **2**.

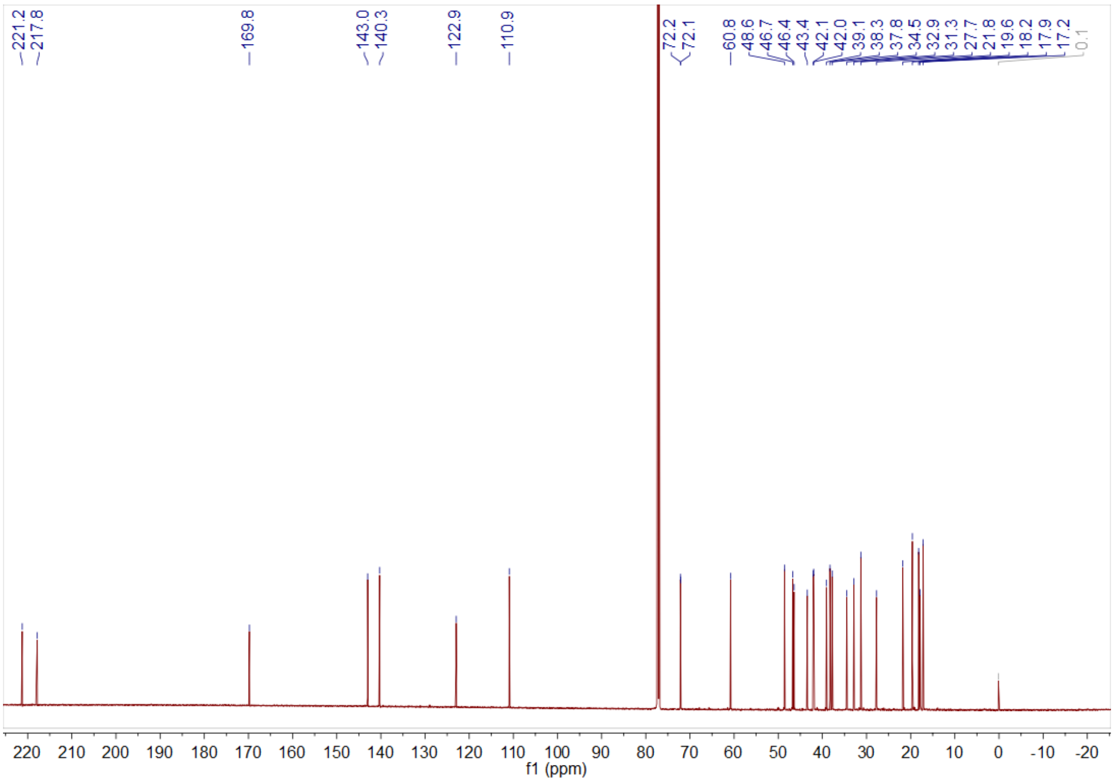

Figure S10. The ^{13}C NMR (150 MHz, $CDCl_3$) spectrum of compound **2**.

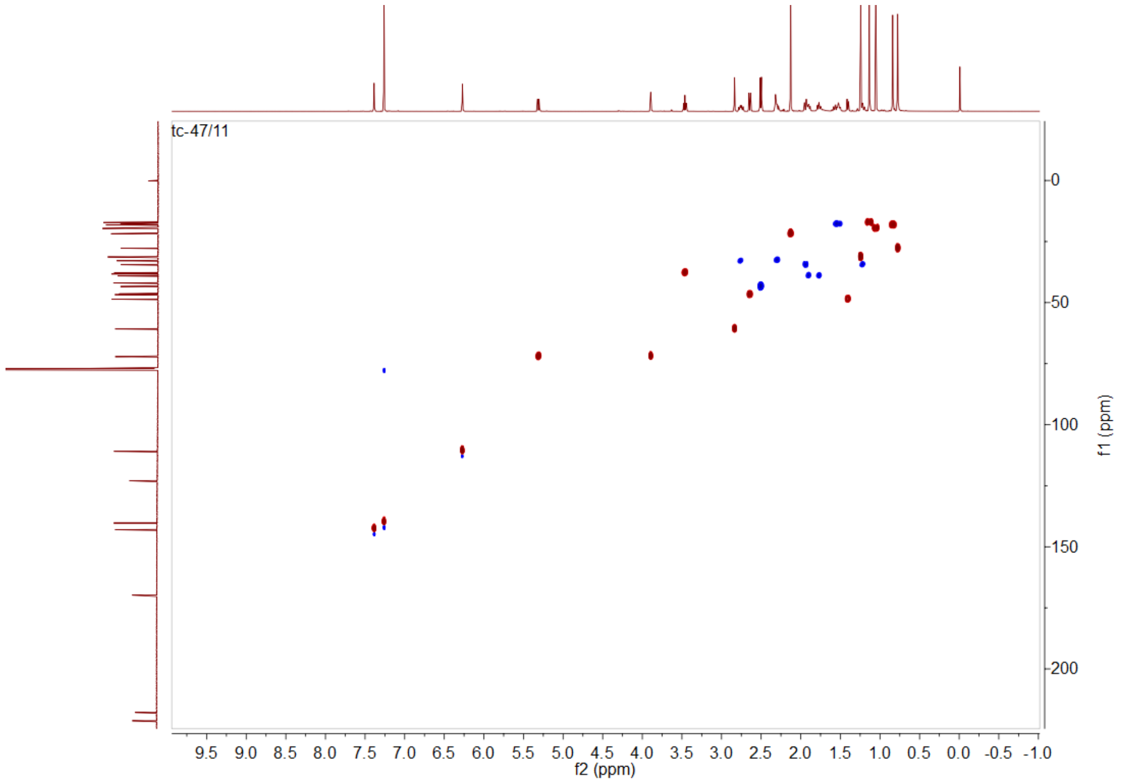

Figure S11. The HSQC ($CDCl_3$) spectrum of compound **2**.

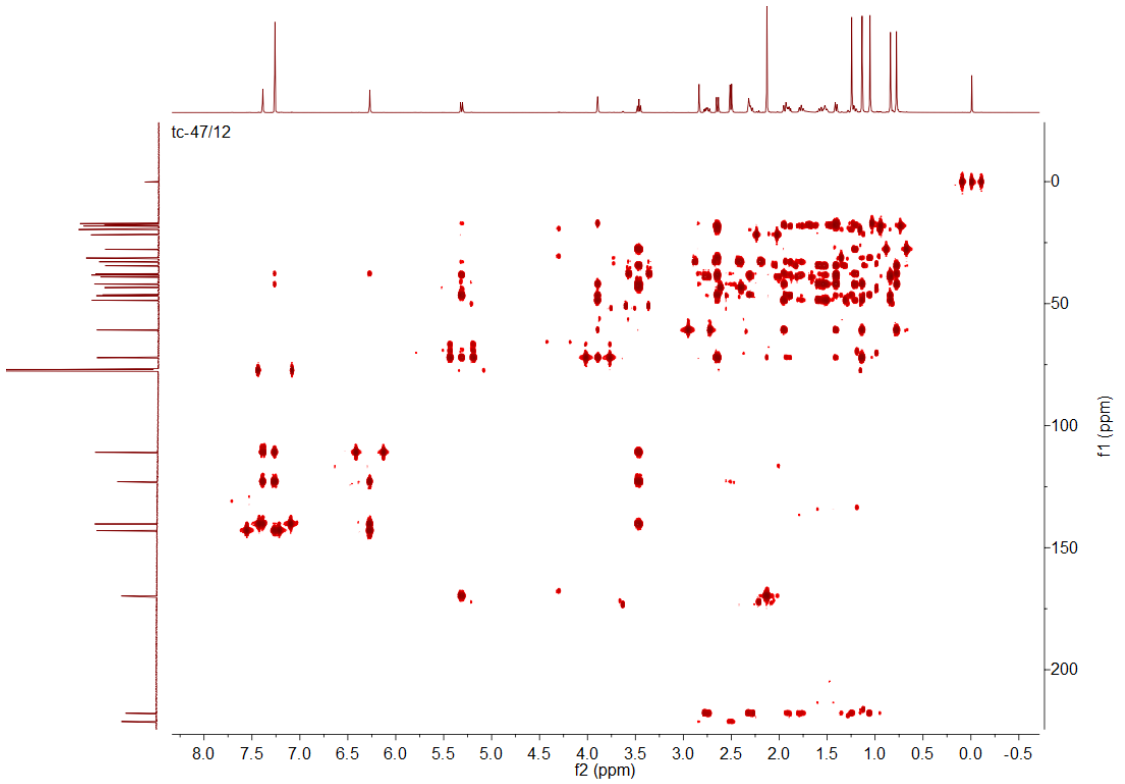

Figure S12. The HMBC ($CDCl_3$) spectrum of compound **2**.

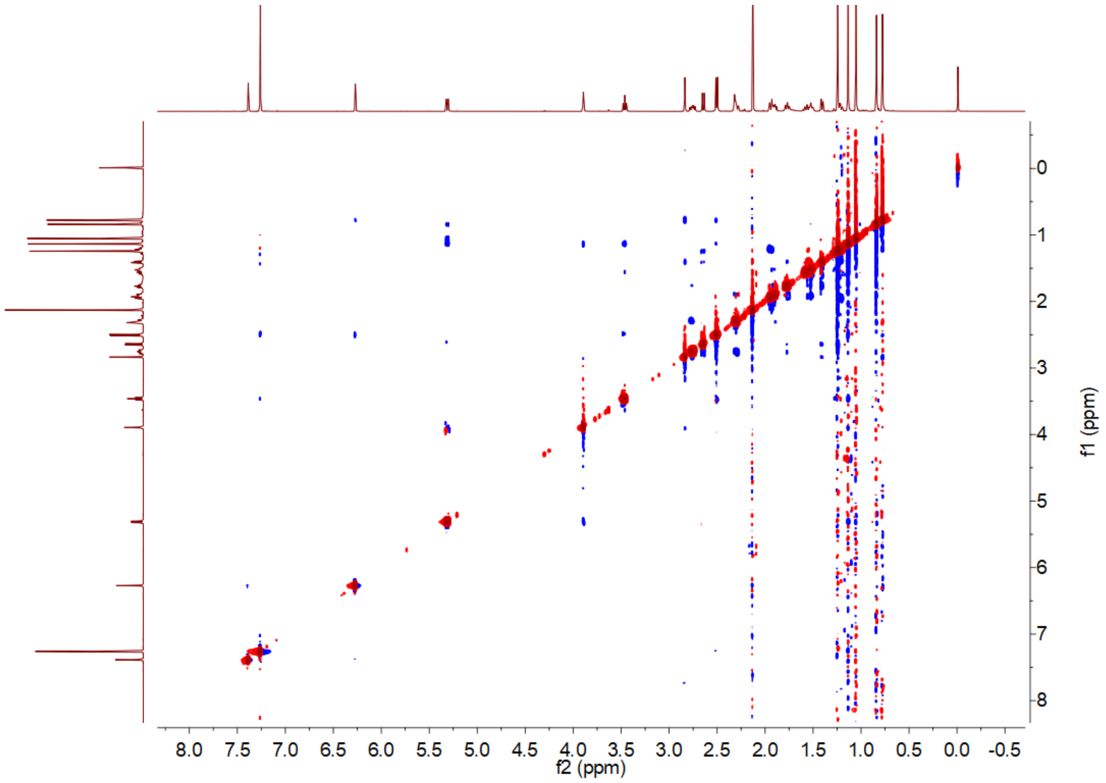

Figure S13. The ROESY ($CDCl_3$) spectrum of compound **2**.

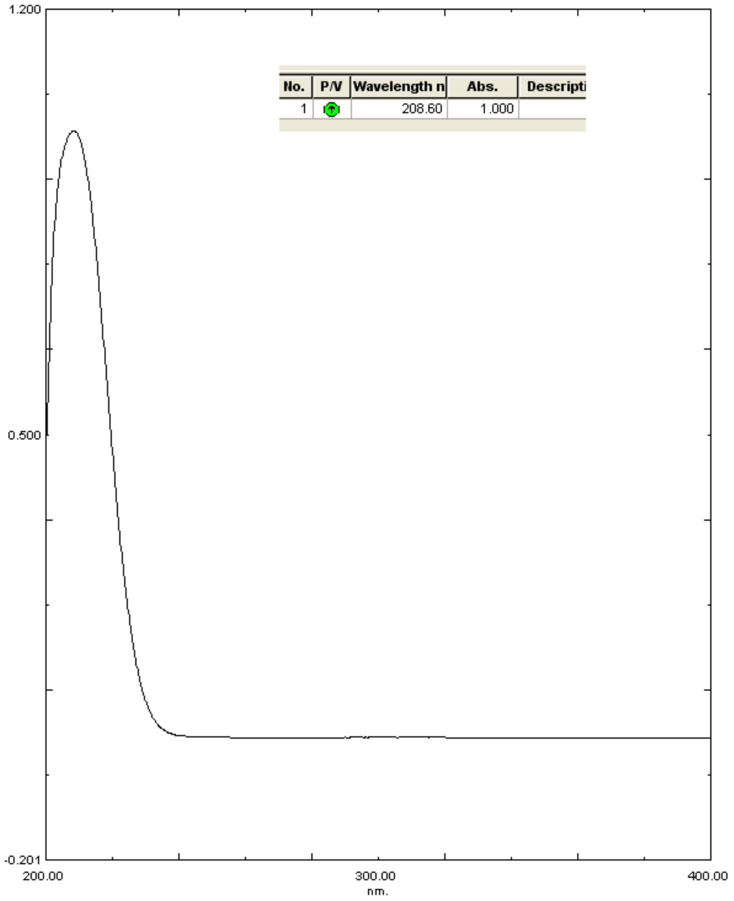

Figure S14. The UV spectrum of compound **2**.

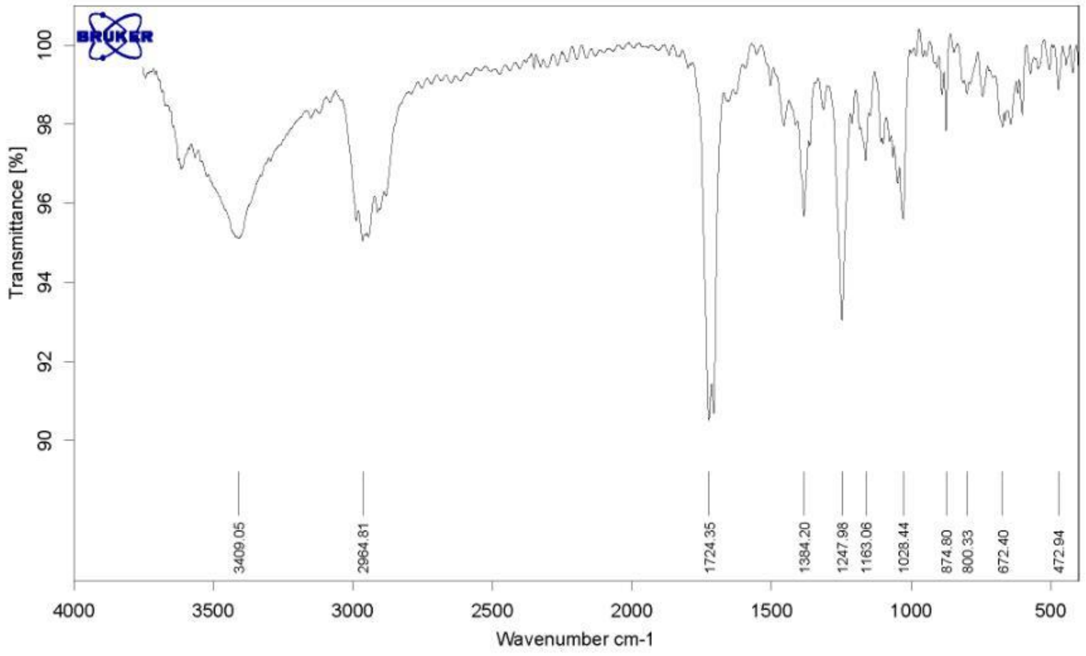

Figure S15. The IR spectrum of compound **2**.

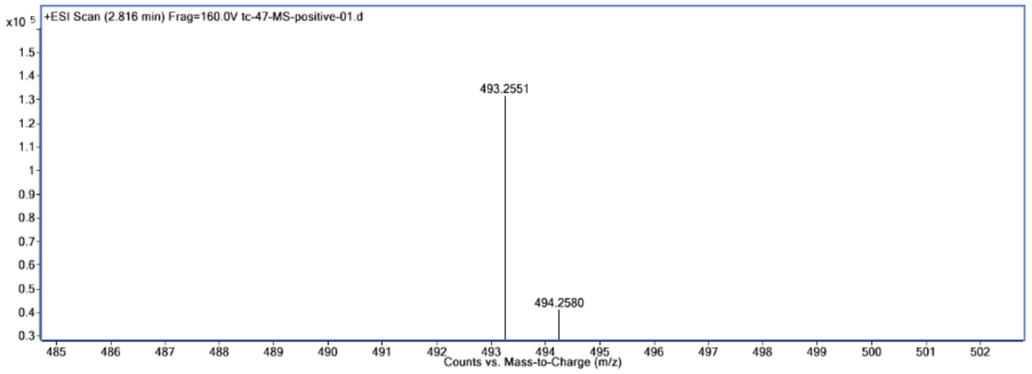

Elemental Composition Calculator

**Target m/z:**	493.2551	**Result type:**	Positive ions	**Species:**	[M+Na]$^+$
**Elements:**		C (0-80); H (0-120); O (0-30); Na (0-5)			
**Ion Formula**		**Calculated m/z**		**PPM Error**	
C28H38NaO6		493.2561		2.02	

Figure S16. The HR-ESI-MS spectrum of compound **2**.

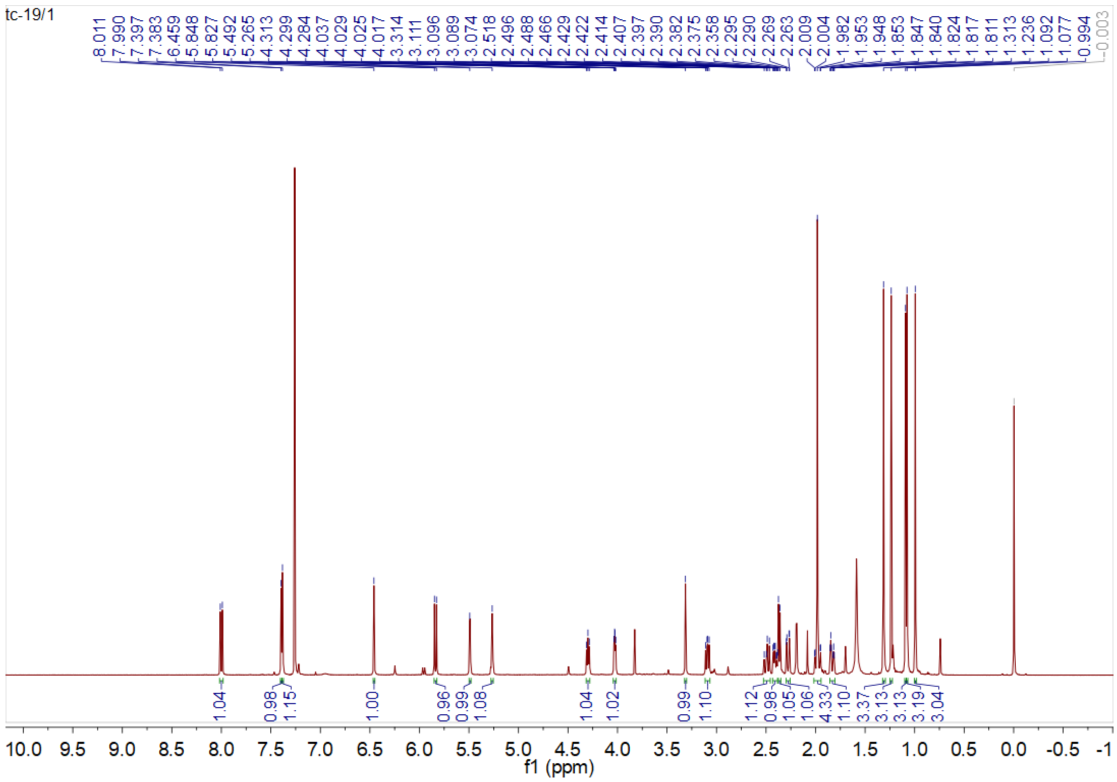

Figure S17. The ^{1}H NMR (500 MHz, $CDCl_3$) spectrum of compound **3**.

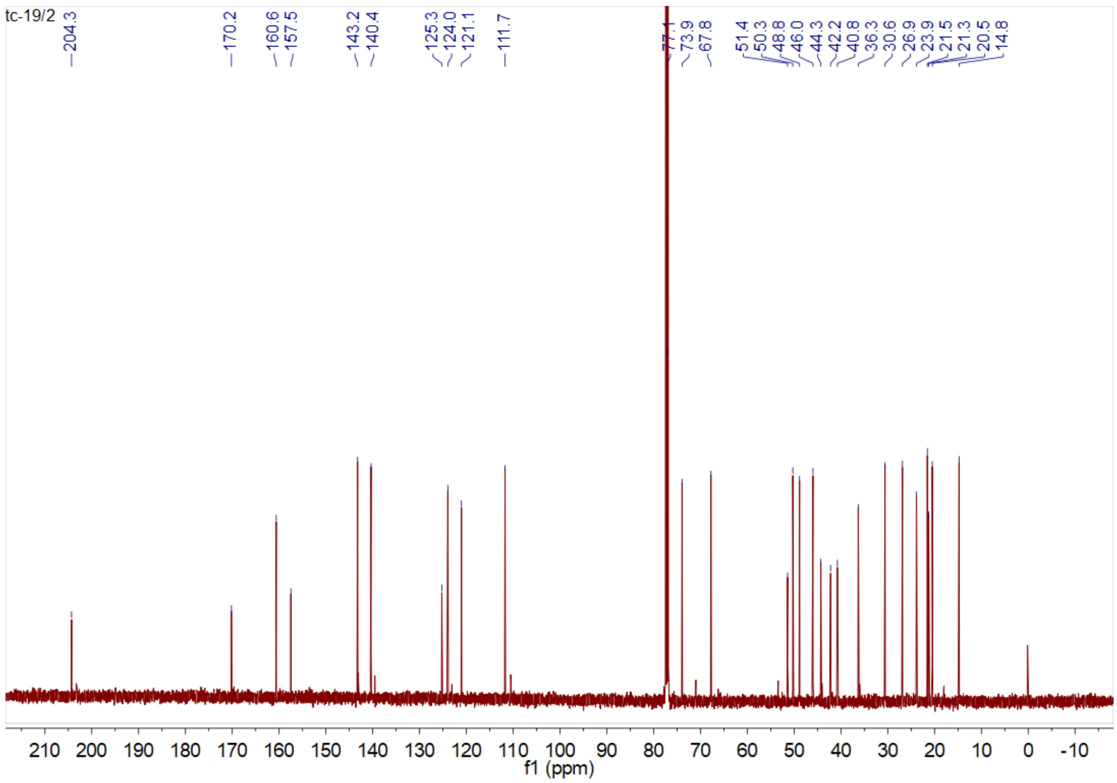

Figure S18. The ^{13}C NMR (125 MHz, $CDCl_3$) spectrum of compound **3**.

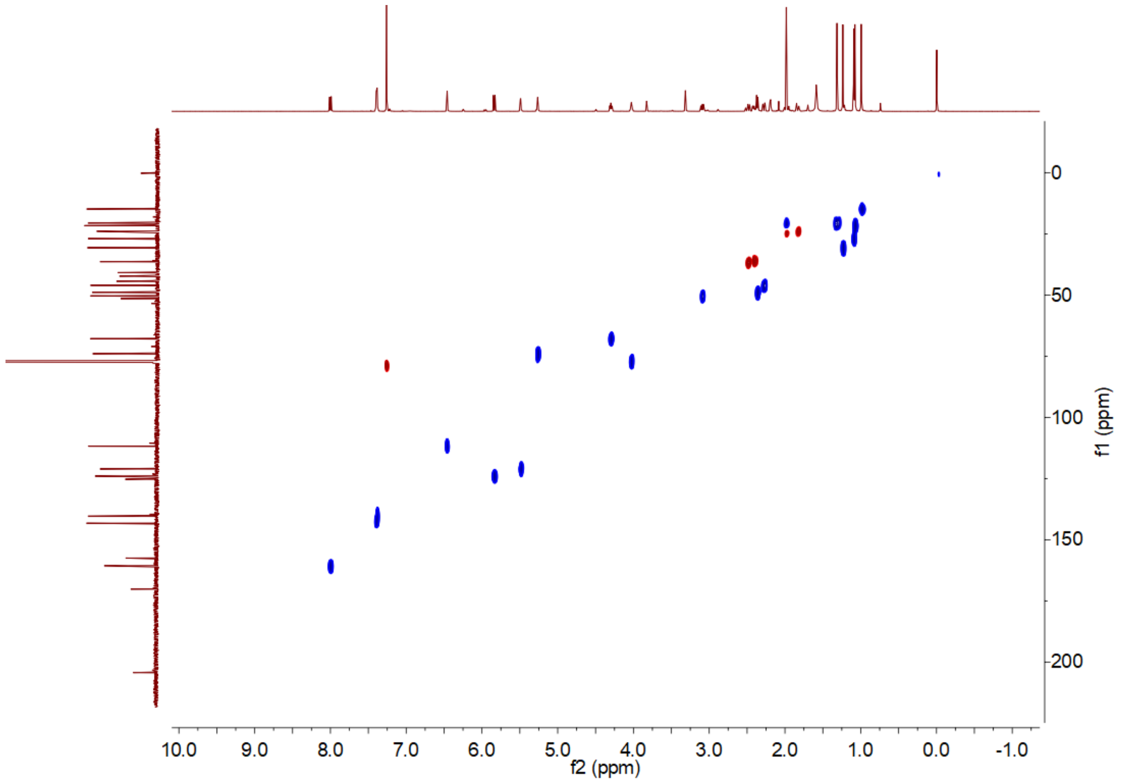

Figure S19. The HSQC ($CDCl_3$) spectrum of compound **3**.

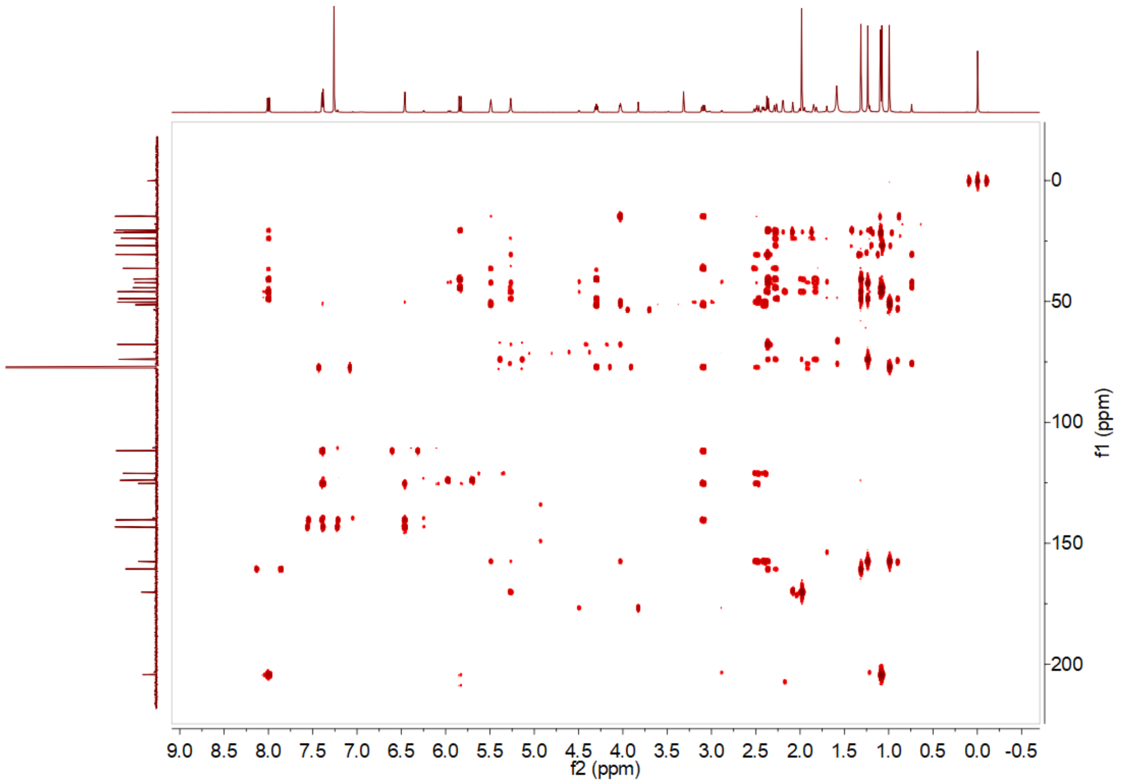

Figure S20. The HMBC ($CDCl_3$) spectrum of compound **3**.

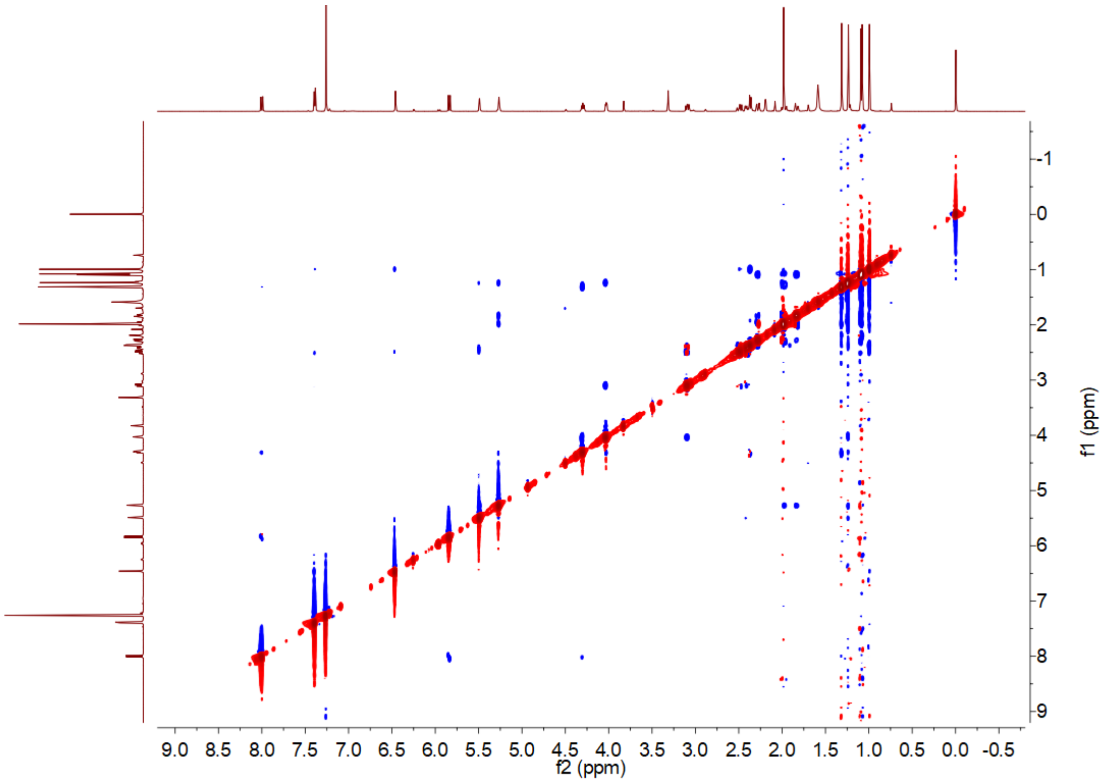

Figure S21. The ROESY ($CDCl_3$) spectrum of compound **3**.

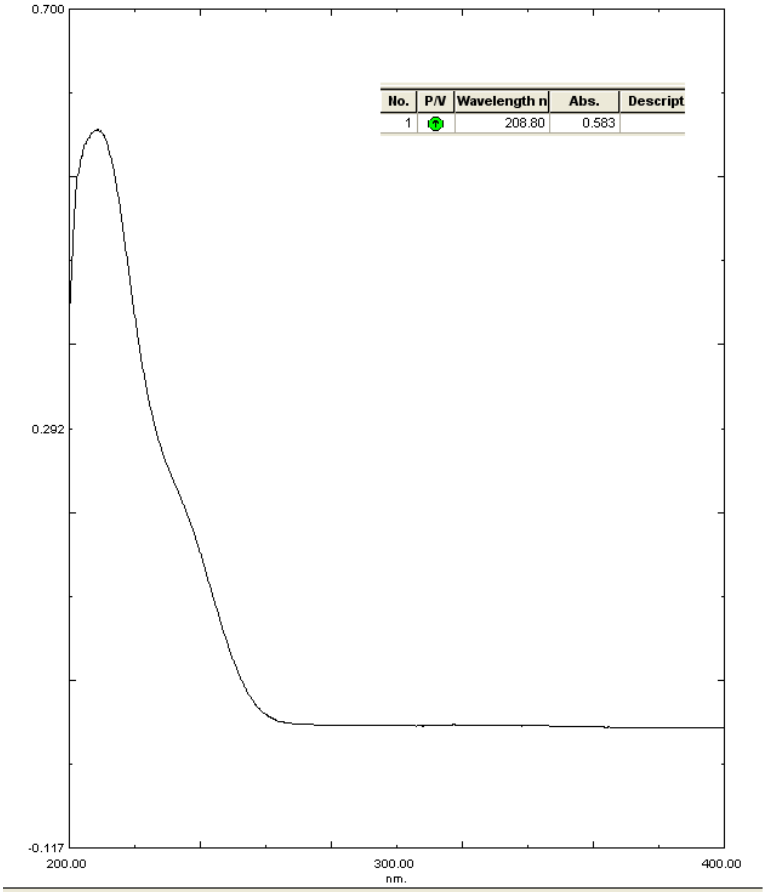

Figure S22. The UV spectrum of compound **3**.

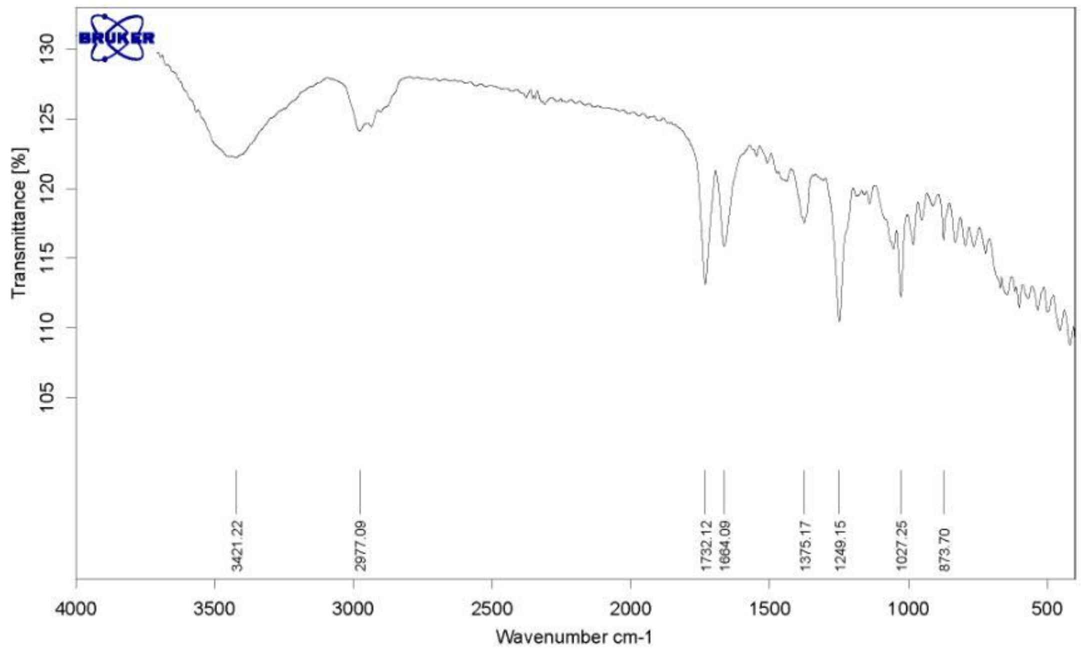

Figure S23. The IR spectrum of compound **3**.

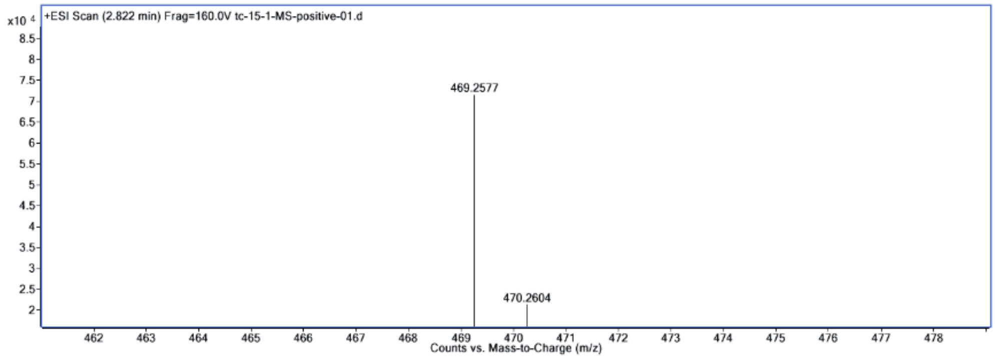

Elemental Composition Calculator

**Target m/z:**	469.2577	**Result type:**	Positive ions	**Species:**	$[M+H]^+$
**Elements:**		C (0-80); H (0-120); O (0-30)			
**Ion Formula**		**Calcalated m/z**		**PPM Error**	
C28H37O6		469.2585		1.66	

Figure S24. The HR-ESI-MS spectrum of compound **3**.

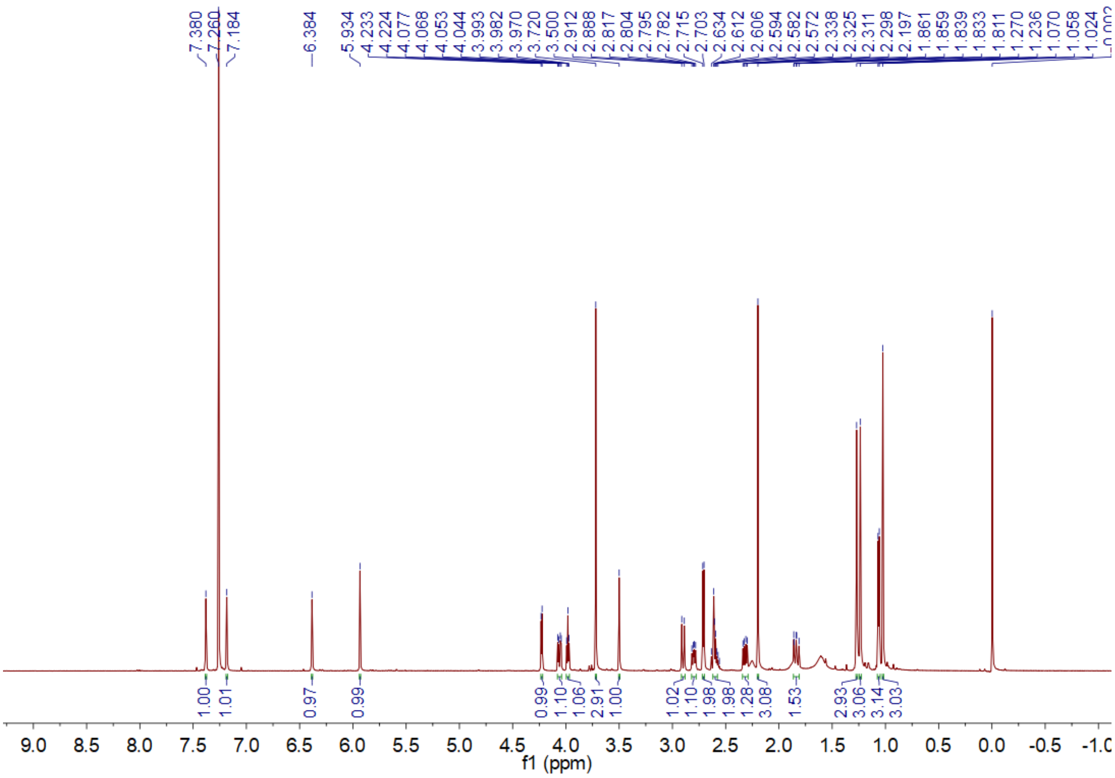

Figure S25. The ^{1}H NMR (500 MHz, $CDCl_3$) spectrum of compound **4**.

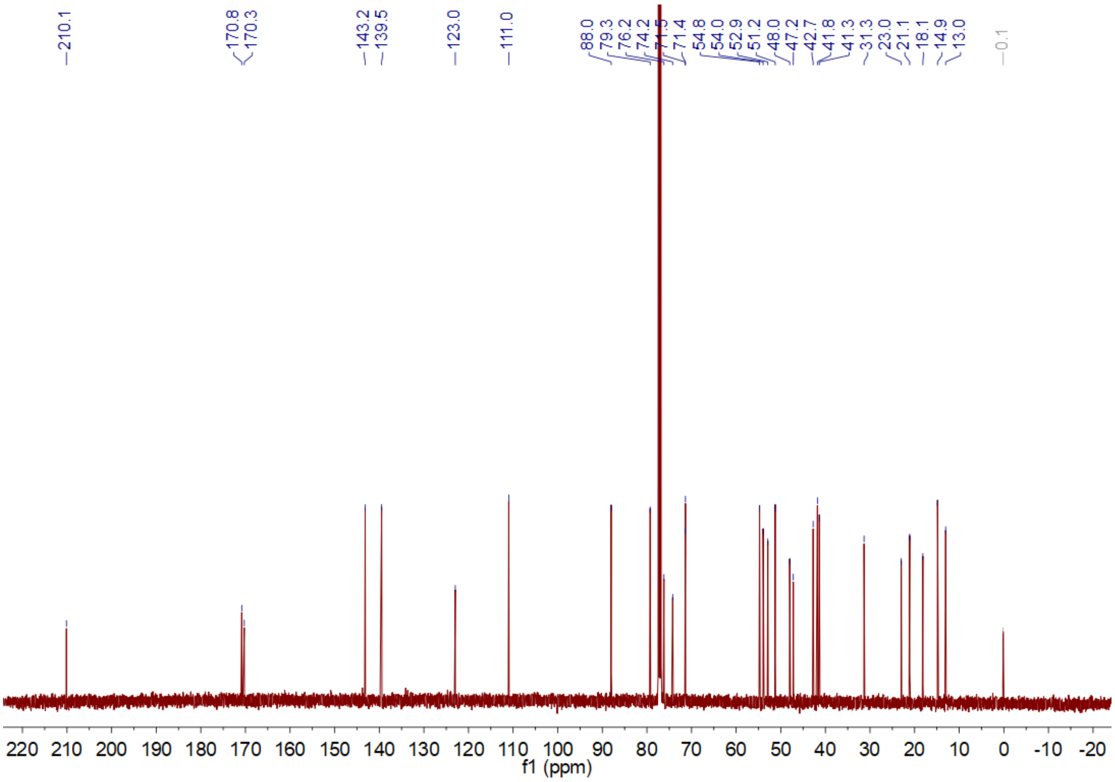

Figure S26. The ^{13}C NMR (125 MHz, $CDCl_3$) spectrum of compound **4**.

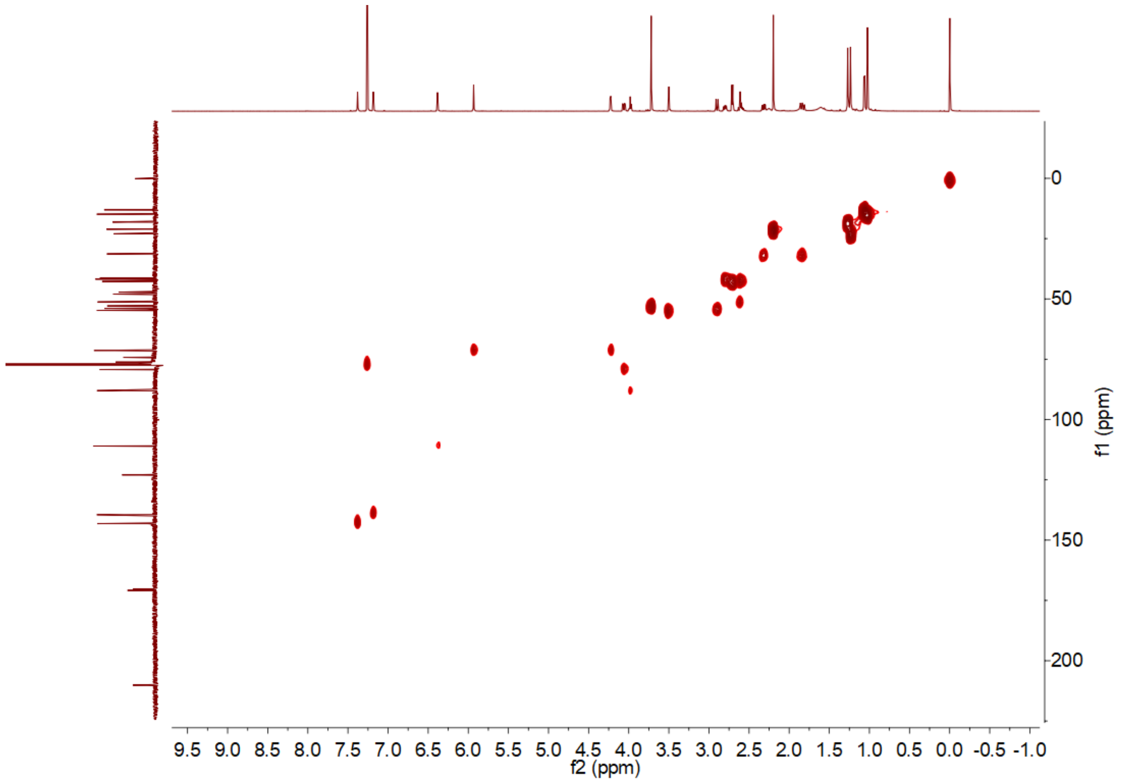

Figure S27. The HSQC ($CDCl_3$) spectrum of compound **4**.

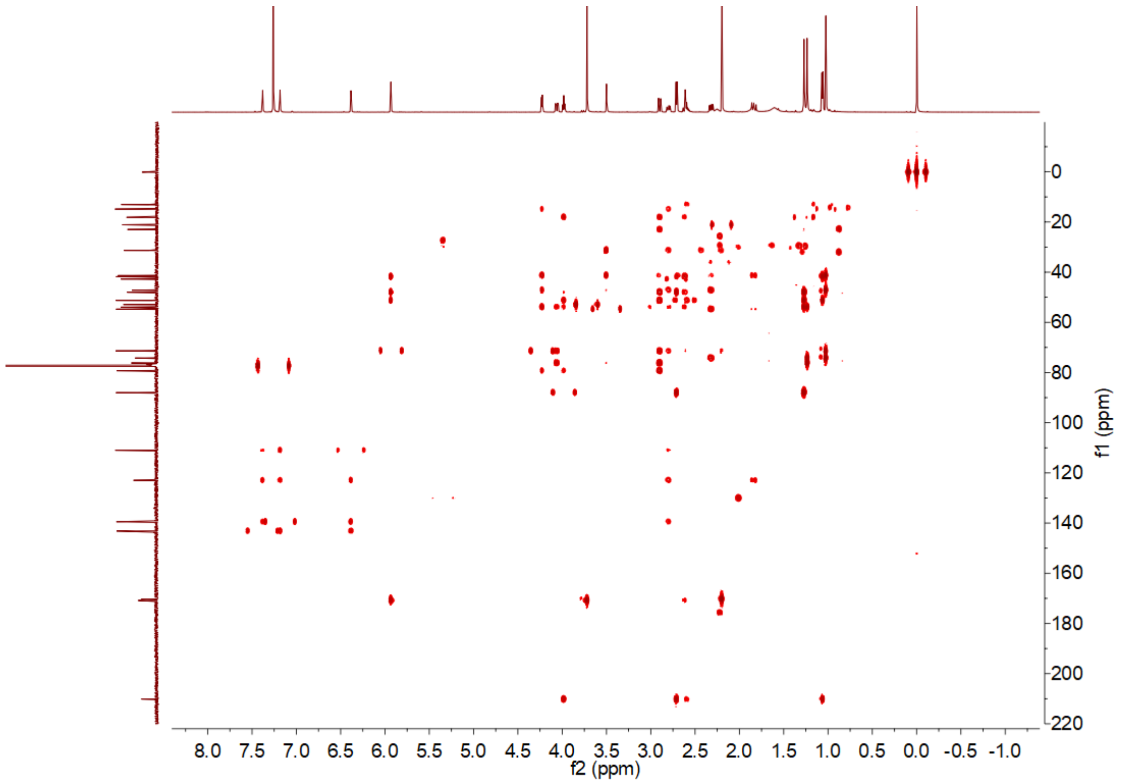

Figure S28. The HMBC ($CDCl_3$) spectrum of compound **4**.

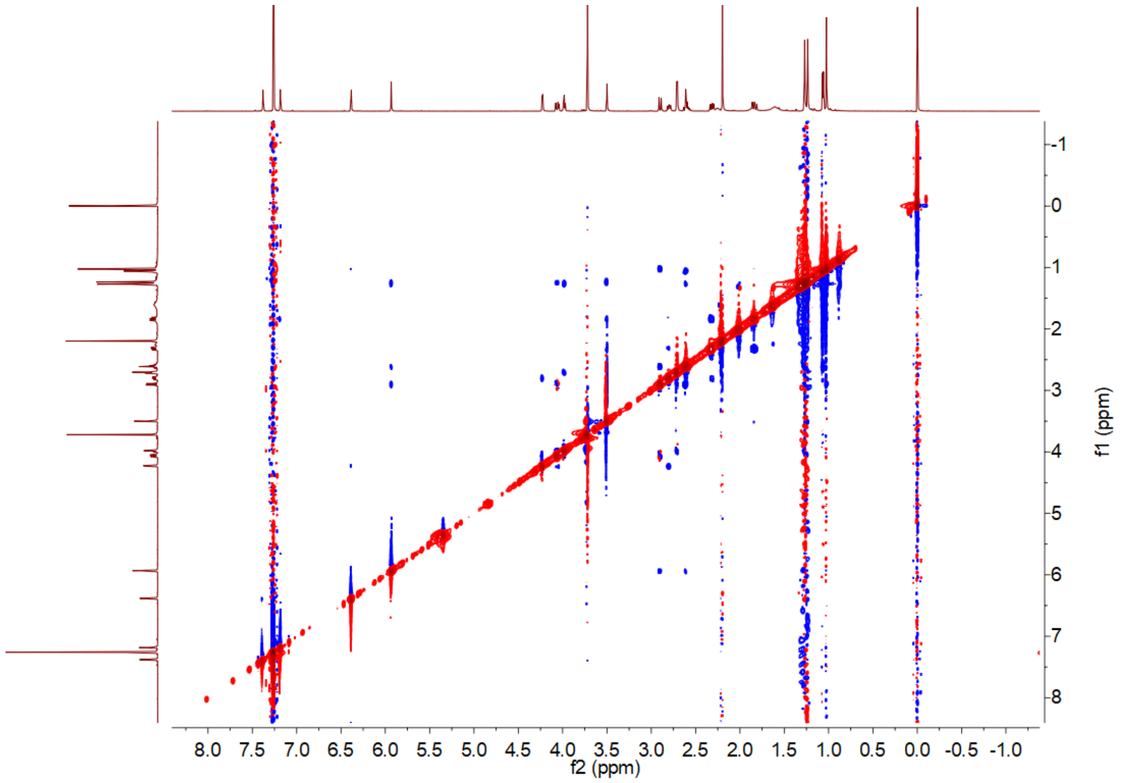

Figure S29. The ROESY ($CDCl_3$) spectrum of compound **4**.

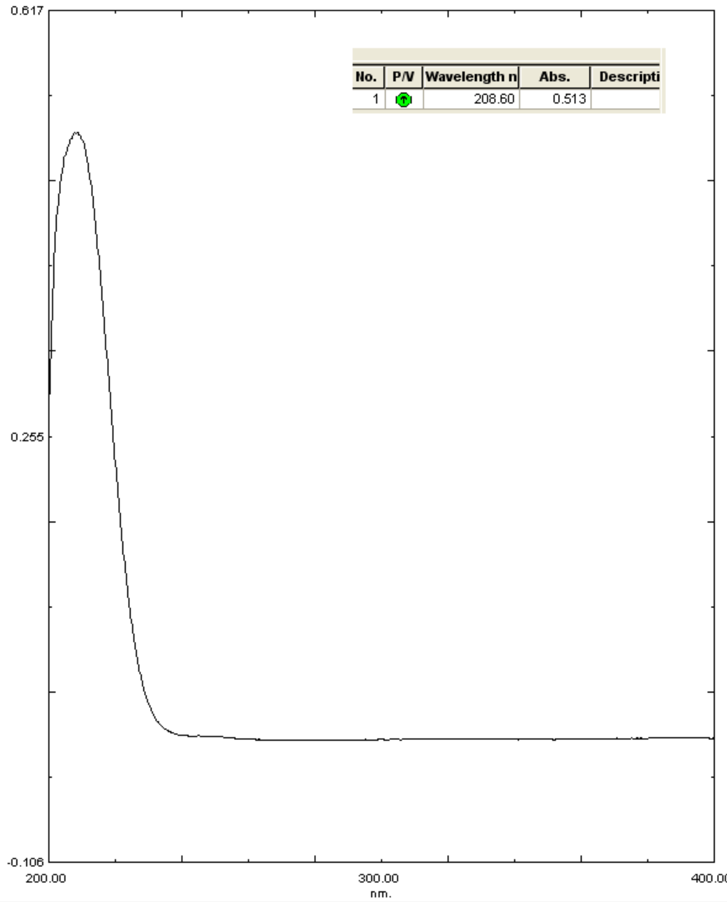

Figure S30. The UV spectrum of compound **4**.

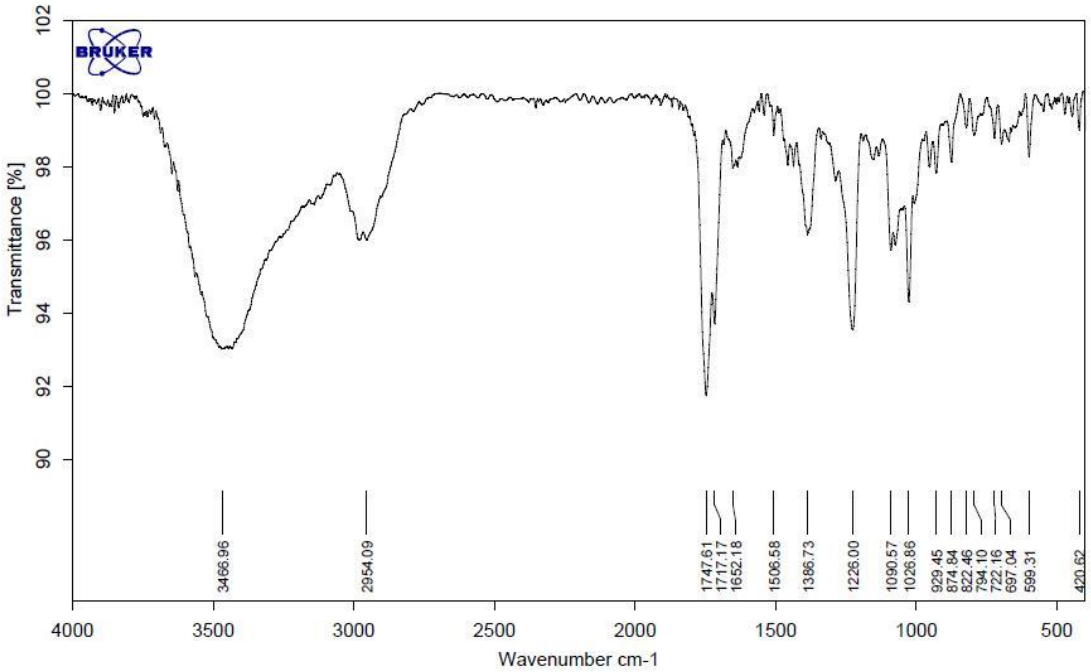

Figure S31. The IR spectrum of compound **4**.

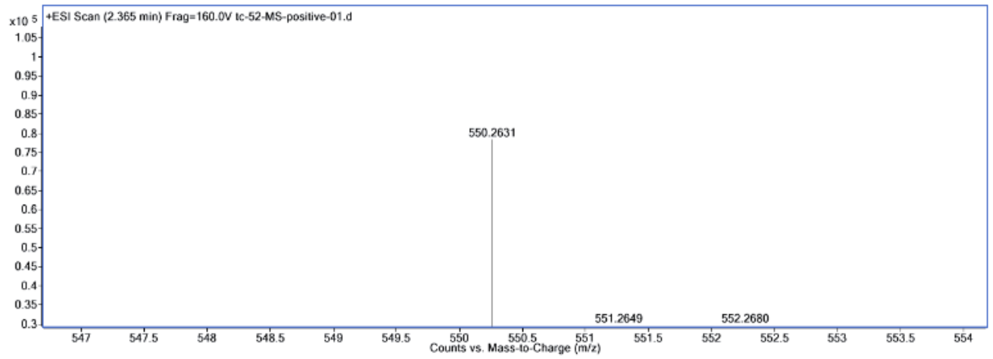

Elemental Composition Calculator

**Target m/z:**	550.2631	**Result type:**	Positive ions	**Species:**	$[M+NH4]^+$
**Elements:**		C (0-80); H (0-120); O (0-30); N(0-10)			
**Ion Formula**		**Calcalated m/z**		**PPM Error**	
C28H40NO10		550.2647		2.79	

Figure S32. The HR-ESI-MS spectrum of compound **4**.
